# Halide Abstraction-Mediated Synthesis of a Highly Twisted Amide

**DOI:** 10.1021/acs.joc.4c01192

**Published:** 2024-08-16

**Authors:** Mizhi Xu, Krista K. Bullard, John Bacsa, Will R. Gutekunst

**Affiliations:** ^†^School of Chemistry and Biochemistry and ^‡^School of Materials Science and Engineering, Georgia Institute of Technology, 901 Atlantic Drive NW, Atlanta, Georgia 30332, United States; § X-ray Crystallography Center, Department of Chemistry, Emory University, 1515 Dickey Drive, Atlanta, Georgia 30322, United States

## Abstract

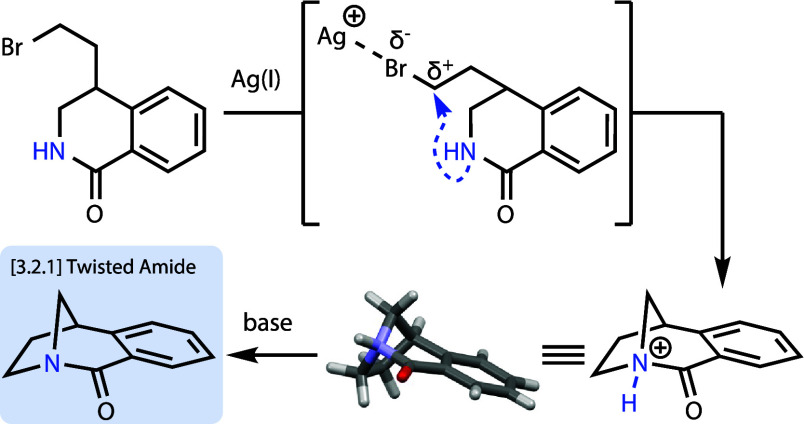

Highly strained [3.2.1] bicyclic twisted amide **2** and corresponding amidium **10** were synthesized for the first time through Ag(I)-mediated halide abstraction. After initial lactamization attempts failed, a second-generation strategy was devised to target amidium **10** via intramolecular alkylation. The 3,4-dihydroisoquinolone cyclization precursor was regioselectively prepared via a Rh(III)-catalyzed C–H activation/annulation reaction. After Ag(I)-assisted cyclization, the [3.2.1] bicyclic framework of amidium **10** was confirmed by X-ray crystallography and enabled full solution characterization of twisted amide **2**.

Amides are normally robust and planar structures that resist hydrolysis due to stabilization through the n_N_–π*_C=O_ resonance. Distortion of the planar amide bonds via electronic, steric, or geometric effects leads to a unique subclass of amides, twisted amides.^1−4^ As a result of the reduced level of amide conjugation, twisted amides feature increased *N*-nucleophilicity and carbonyl electrophilicity and thus behave more like “amino ketones” than traditional planar amides. As demonstrated by a number of experimental and computational studies, amide distortion increases the reactivity of twisted amides, which can potentially lead to undesired side reactions during synthesis and isolation. Since Lukeš first proposed the distorted amide bond in [2.2.1] and [2.2.2] bicyclic structures in 1938,^5^ a variety of cyclization strategies have been developed to synthesize bicyclic twisted amides, including direct condensation reactions,^6−13^ Schmidt–Aubé reactions,^14−18^ Diels–Alder reactions,^19−21^ Heck reactions,^22−26^ carbene insertion reactions,^27−30^ and other methods.^1,31,32^

Recently, we have introduced a new class of living polymerization based on the ability of nucleophilic twisted amides to undergo alkylation/halide-rebound processes.^13,33^ While it was found that remote electron-donating groups could increase both amide distortion and *N*-nucleophilicity in a benzo-fused [3.3.1] twisted amide system **1** (Figure 1A), interest was directed toward further enhancing the twisted amide reactivity via modifying geometric parameters. Instead of substitution, the [3.2.1] analogue of **2** was targeted, which removes one carbon atom in the bicyclic skeleton (Figure 1A). This modification leads to a more strained framework and a more twisted amide bond, as shown by Brown.^10,34^ To compare the degree of distortion of **1** and **2**, the Winkler–Dunitz parameters of twist angle τ and *N*-pyramidalization χ_N_ were calculated, leading to the additive Winkler–Dunitz parameter τ + χ_N_ that accurately correlates to amide distortion.^35,36^ The computationally optimized ground state geometry revealed that the τ + χ_N_ value (87.4° vs 118.0°) significantly increased as a result of this geometric modification (Figure 1A). However, synthesis and isolation of highly twisted amides have been challenging due to their propensity for hydrolysis or polymerization.^27,28^ To date, only a handful of [3.2.1] bicyclic twisted amides have been successfully synthesized, and most amides are embedded in complex multicyclic frameworks (Figure 1B).^6,21,30,37−39^ These examples of bridged highly twisted amides required the multistep synthesis of precursors and have regioselectivity issues in the key cyclization step. To produce twisted amide **2**, two pathways were proposed after considering the different retrosynthetic disconnections (Figure 1C). Pathway **a** involves the formation of the amide bond from a pyrrole precursor, which follows the strategy developed for the less strained [3.3.1] twisted amide **1**.^13^ The second approach involves directly twisting a planar amide through an intramolecular alkylation strategy starting from a 3,4-dihydroisoquinolone precursor (pathway **b**).^38^ Herein, these two straightforward strategies are detailed for the synthesis of [3.2.1] bridged twisted amide **2**.

**Figure 1:**
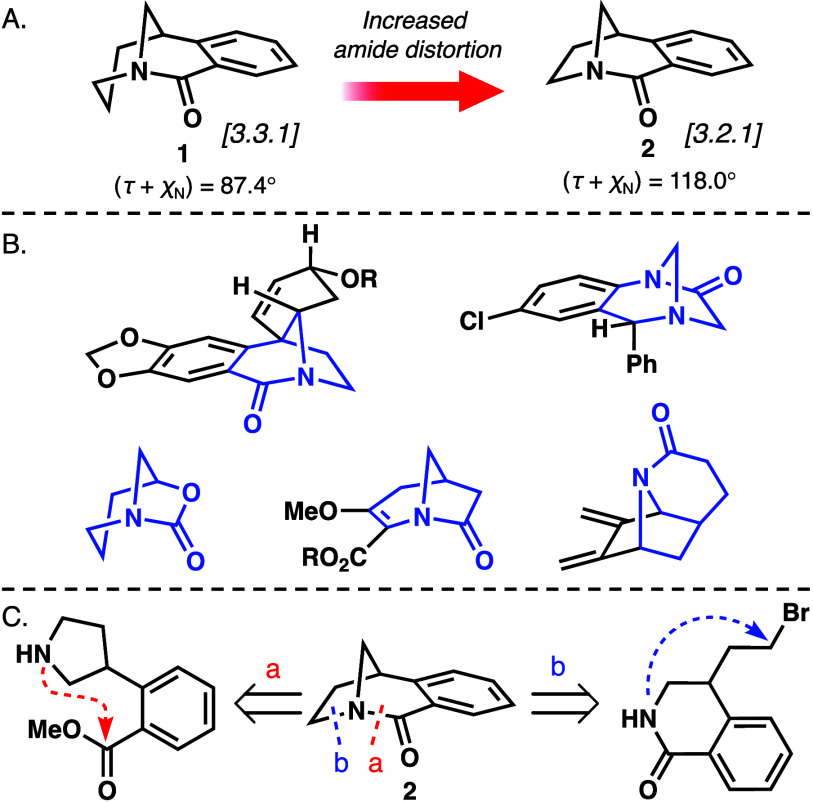
(A) Structures and calculated additive Winkler–Dunitz parameters of twisted amides **1** and **2**. (B) Reported [3.2.1] bicyclic twisted amide frameworks. (C) Retrosynthetic analysis of **2**.

Initial attempts to prepare twisted amide **2** through pathway a were investigated starting from commercially available pyrrolidinium salt **3**. Upon treatment with cesium carbonate in acetonitrile at a concentration of 0.01 M at room temperature, clean conversion to a new product was observed by ^1^H nuclear magnetic resonance (NMR) after 30 min. However, further examination of the reaction mixture by NMR and GC-MS suggested it was only free amine **4** (Scheme 1 and Figure S1). Heating the reaction mixture to 60 °C under these conditions led to cyclization to form twisted amide **2**, as evidenced by NMR and GC-MS analyses. The use of 1,8-diazabicyclo[5.4.0]undec-7-ene (DBU) as a base at a higher concentration of **3** (0.1 M) also produced **2** within 2 h at room temperature (Figure S2). The conversion to **2** was further supported by an *in situ* reaction with excess iodomethane. The isolation of halide-rebound ring-opening product **6** in 53% yield via intermediate methyl amidium **5** supports the generation of **2** from direct lactamization (Scheme 1).

**Scheme 1:**
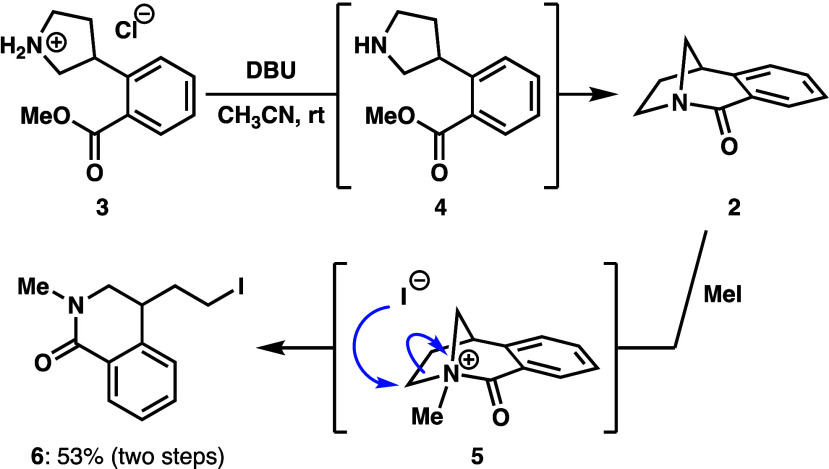
Cyclization of Pyrrolidinium **3** to Synthesize Twisted Amide **2** and Subsequent Formation of **6** via the Halide-Rebound Reaction

Although the formation of twisted amide **2** was observed in solution, stirring the reaction mixture for extended periods of time eventually led to broadened peaks in the ^1^H NMR spectrum, likely due to the oligomerization of **2** under the reaction conditions (Figure S2). The instability of **2** prevented the successful isolation of the pure twisted amide through column chromatography or filtration. Even rotary evaporation of the solution containing twisted amide **2** resulted in an insoluble residue, likely due to oligomerization and/or hydrolysis. This suggests that twisted amide **2** is persistent only in a moderately dilute solution due to intermolecular reactions. The oligomerization of a highly twisted amide has also been observed by Stoltz and co-workers during their attempt to neutralize the protonated [2.2.2] bicyclic 2-quinoclidone.^15^ Given the unsuccessful isolation of twisted amide **2** with pathway **a**, synthetic pathway **b** was then examined.

To explore alkylation strategy b, a route to the bromide precursor, 4-(2-bromoethyl)-3,4-dihydroisoquinolone **7**, was needed. This was achieved with an established Rh(III)-catalyzed C–H activation/annulation reaction between a benzohydroxamate derivative and 4-bromo-1-butene.^40−43^ When linear α-olefins are used in this reaction, the regioselectivity of annulation is known to be low. Indeed, the reaction between *O*-pivaloyl benzohydroxamate and 4-bromo-1-butene using the [Cp*RhCl_2_]_2_ catalyst (Cp* = 1,2,3,4,5-pentamethylcyclopentadienyl ligand) resulted in nearly equal amounts of **7** and **8** (Scheme 2). Gratifyingly, when the more sterically hindered Rh(III) catalyst **9** reported by Perekalin and co-workers was used in the reaction between *O*-Boc benzohydoxamate and 4-bromo-1-butene, the desired product **7** was obtained in 79% yield with complete regioselectivity (Scheme 2).^44^ Here, switching the protecting group from pivaloyl to Boc is essential for highly regioselective annulation. Notably, this synthesis of **7** could be readily scaled up to half-gram quantities, enabling subsequent tests of the cyclization step in strategy **b**.

**Scheme 2:**
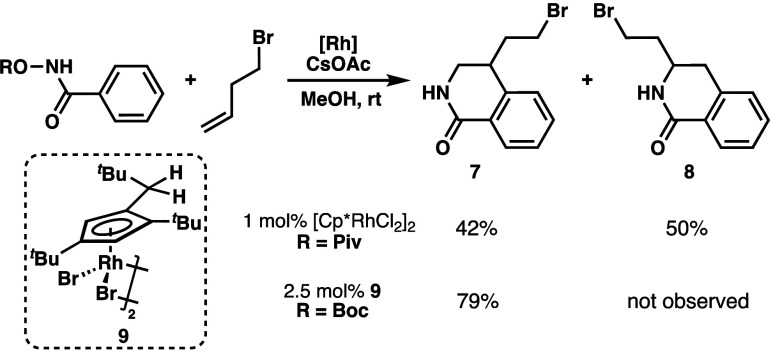
Synthesis of 3,4-Dihydroisoquinolone **7**

With precursor **7** prepared, cyclization experiments were performed to produce [3.2.1] twisted amide **2**. In a recent report on the total synthesis of gracilamine, a chlorine-containing 3,4-dihydroisoquinolone intermediate was successfully converted into a [3.2.1] twisted amide.^38^ In that synthesis, deprotonation of the amide nitrogen by NaH induced the intramolecular substitution of alkyl chloride to generate the twisted amide. Unfortunately, when **7** was mixed with NaH, only broad NMR peaks were observed, indicating the formation of **2** followed by *in situ* oligomerization (Figure S3).

Due to the unsuccessful preparation of twisted amide **2** under basic conditions, focus was directed toward the synthesis and isolation of its conjugate acid, amidium **10** (Figure 2). As demonstrated by Stoltz, protonation of the nucleophilic nitrogen atom in highly strained twisted amides can enhance the stability of amide bonds under anhydrous conditions.^15^ A silver-assisted halide abstraction approach was proposed for the cyclization on the basis of the high affinity between the silver(I) cation and halides, which has been well-documented to promote the nucleophilic substitution of alkyl halides.^45^

**Figure 2:**
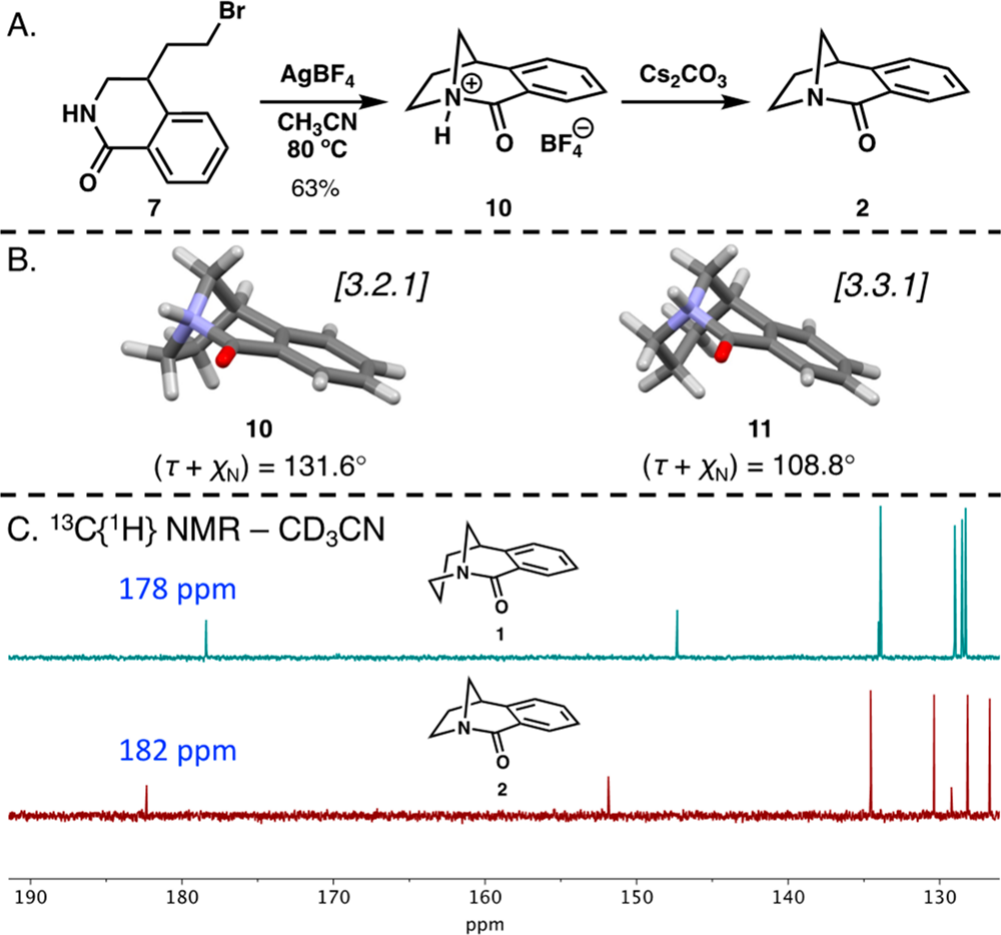
(A) Synthesis of amidium **10** and amide **2**. (B) Crystal structures of amidiums **10** and **11** (counterions have been omitted for the sake of clarity; ellipsoid contour at 50% probability). (C) Stacked ^13^C{^1^H} NMR spectra of twisted amides **1** and **2**.

The cyclization tests were then performed on a 50 mg scale. When bromide **7** was treated with AgBF_4_ in acetonitrile at 80 °C, 4-(2-bromoethyl)-3,4-dihydroisoquinolone **7** was smoothly transformed into amidium **10** according to ^1^H NMR spectroscopy. The amidium could be isolated when carefully precipitated from anhydrous diethyl ether in 63% yield (Figure 2A). Recrystallization of amidium **10** using anhydrous dichloromethane and diethyl ether permitted an unambiguous characterization by X-ray crystallography. The τ + χ_N_ value of [3.2.1] amidium **10** (131.6°) was higher than that of [3.3.1] amidium **11** (108.8°), supporting the more distorted N–C(O) bond due to enhanced geometric constraints (Figure 2B).

The isolation of parent twisted amide **2** via deprotonation of **10** was attempted; however, both rapid column chromatography and filtration led to decomposition and insoluble products. Despite the failure to isolate **2**, *in situ* neutralization of **10** in solution using the inorganic base Cs_2_CO_3_ did not lead to oligomerization and enabled the solution characterization of pure **2** via NMR spectroscopy. Notably, the ^1^H NMR spectrum of **2** was consistent with the spectrum obtained in pathway a during cyclization of **3** under basic conditions (Figure S4), further suggesting that both pathways can lead to the formation of twisted amide **2**. In addition, the ^13^C NMR spectrum of **2** clearly revealed a carbonyl chemical shift at 182 ppm that is downfield compared to the carbonyl shift of [3.3.1] analogue **1** (178 ppm), suggesting reduced amide resonance and enhanced distortion of amide **2** (Figure 2C).

In conclusion, the first synthesis of [3.2.1] bicyclic twisted amide **2** was reported via two distinct pathways. First, cyclization of pyrrolidinium salt **3** led to the formation of **2** in solution, as supported by the *in situ* reaction with MeI to form alkyl iodide **6**. Further purification and isolation of **2**, though, were unsuccessful due to its inherent reactivity. Through the use of a silver(I)-assisted cyclization, planar amide **7** could be coerced into cyclization to give stable amidium **10**, which was successfully isolated and characterized through X-ray crystallography. The expeditious preparation of twisted amide precursors via a Rh(III)-mediated C–H activation/annulation chemistry in conjunction with straightforward silver(I)-promoted cyclization holds promise for accessing other challenging amide frameworks in the future.

## Experimental Section

### General Information

All reactions were carried out under a nitrogen atmosphere with dry solvents using anhydrous conditions, unless otherwise stated. Dry, degassed tetrahydrofuran (THF), acetonitrile (CH_3_CN), and dichloromethane (DCM) were obtained from a JC Meyer solvent purification system. Anhydrous acetonitrile and diethyl ether used in the syntheses and crystal growth of amidiums **10** and **11** were purchased from Acros Organics. Pyrrolidinium hydrochloride **3** was purchased from Sigma-Aldrich. Unless otherwise stated, all other reagents were purchased at the highest commercial quality and used without further purification. Yields refer to chromatographically and spectroscopically (^1^H NMR) homogeneous materials unless otherwise stated. Reactions were monitored by thin layer chromatography (TLC) carried out on 0.25 mm E. Merck silica gel plates (60F-254) using ultraviolet light as the visualizing agent and basic aqueous potassium permanganate (KMnO_4_) and heat as the developing agents. Silica gel [60 Å, 40–63 μm (230–400 mesh)] from Silicycle was used for flash column chromatography. NMR spectra were recorded on Bruker Avance 400 or 500 MHz instruments, calibrated using residual undeuterated solvent as an internal reference (CHCl_3_ at 7.26 ppm for ^1^H NMR and 77.16 ppm for ^13^C NMR, CH_3_CN at 1.94 ppm for ^1^H NMR and 1.32 ppm for ^13^C NMR, and DMSO at 2.50 ppm for ^1^H NMR and 39.52 ppm for ^13^C NMR), and then analyzed by MestReNova. The following abbreviations (or combinations thereof) were used to explain the multiplicities: s, singlet; d, doublet; t, triplet; q, quartet; m, multiplet; br, broad. Mass spectra (MS) were recorded on a GC-MS instrument (Agilent Technologies 7890A GC system/5975C with Triple-Axis Detector). High-resolution mass spectra (HRMS) were recorded via ESI by using a Thermo IDX mass spectrometer or a Thermo Scientific QEHF mass spectrometer. Melting points were measured on a MEL-TEMP II Laboratory Devices instrument (uncorrected).

### Synthesis and Characterization

#### *In Situ* Formation of **2** from **3** and Subsequent Synthesis of **6**

Under N_2_, to a solution of pyrrolidinium salt **3** (24.2 mg, 0.1 mmol, 1 equiv) in anhydrous acetonitrile (1 mL, 0.1 M for **3**) in an 8 mL vial was added DBU (18.3 mg, 18 μL, 0.12 mmol, 1.2 equiv). The reaction mixture was stirred at room temperature for 2 h, and aliquots of the reaction mixture were taken for ^1^H NMR and GC-MS analyses. The formation of twisted amide **2** was observed on the basis of ^1^H NMR and GC-MS (*m*/*z* 173.1).

To the reaction vial was then added MeI (141.9 mg, 62 μL, 1.0 mmol, 10 equiv). The reaction mixture was stirred in a preheated oil bath at 80 °C overnight, before being concentrated on a rotary evaporator. The residue was purified by column chromatography on silica gel (50% EtOAc/hexanes) to give compound **6** (colorless oil, 17.3 mg, 53% for two steps): ^1^H NMR (500 MHz, CDCl_3_, 298 K) δ 8.09 (dd, *J* = 7.7, 1.3 Hz, 1H), 7.44 (td, *J* = 7.5, 1.4 Hz, 1H), 7.37 (td, *J* = 7.6, 1.2 Hz, 1H), 7.27 (d, *J* = 7.5 Hz, 1H), 3.88 (dd, *J* = 12.7, 4.3 Hz, 1H), 3.26 (dd, *J* = 12.7, 2.4 Hz, 1H), 3.19 (dt, *J* = 10.1, 6.4 Hz, 1H), 3.15 (s, 3H), 3.10–3.03 (m, 2H), 2.25–2.06 (m, 2H); ^13^C{^1^H} NMR (126 MHz, CDCl_3_, 298 K) δ 164.4, 140.2, 131.8, 128.8, 128.7, 127.8, 126.9, 52.4, 37.8, 37.2, 35.6, 4.3; HRMS (ESI) *m*/*z* [M + H]^+^ calcd for C_12_H_15_INO 316.0193, found 316.0203.

#### Synthesis of **7** Using [Cp*RhCl_2_]_2_

The *O*-Piv^41^ and *O*-Boc^43^ benzohydoxamates were prepared according to literature methods. The procedure using [Cp*RhCl_2_]_2_ was modified from literature methods.^41,46^ Without any particular precaution to extrude oxygen or moisture, *O*-Piv benzohydoxamate (442.5 mg, 2 mmol, 1 equiv), [Cp*RhCl_2_]_2_ (12.4 mg, 0.02 mmol, 0.01 equiv), and cesium acetate (96.0 mg, 0.5 mmol, 0.25 equiv) were added in a 20 mL vial equipped with a stir bar. MeOH (10 mL) was then added followed immediately by 4-bromo-1-butene (297.0 mg, 223 μL, 2.2 mmol, 1.1 equiv). The reaction mixture was stirred at room temperature for 16 h. After the reaction had reached completion, the mixture was diluted with EtOAc and passed through a short pad of silica with EtOAc as the eluent. The filtrate was concentrated, and the residue was purified by column chromatography on silica gel (20% to 67% EtOAc/hexanes) to give products **8** (less polar, 260.7 mg, 50%) and **7** (more polar, 221.4 mg, 42%). The structures of **7** and **8** were determined by DEPT 135 experiments.

**7**: pale yellow solid; mp 118–119 °C; ^1^H NMR (400 MHz, CDCl_3_, 298 K) δ 8.09 (dd, *J* = 7.7, 1.5 Hz, 1H), 7.51 (td, *J* = 7.5, 1.5 Hz, 1H), 7.41 (td, *J* = 7.6, 1.3 Hz, 1H), 7.34 (d, *J* = 7.5 Hz, 1H), 6.02 (s, 1H), 3.84 (dd, *J* = 12.6, 4.2 Hz, 1H), 3.51–3.43 (m, 1H), 3.38 (ddd, *J* = 12.6, 5.0, 2.2 Hz, 1H), 3.31 (ddd, *J* = 10.4, 8.4, 5.2 Hz, 1H), 3.26–3.16 (m, 1H), 2.36–2.13 (m, 2H); ^13^C{^1^H} NMR (126 MHz, CDCl_3_, 298 K) δ 166.4, 141.2, 132.4, 128.4, 128.3, 127.7, 127.4, 44.1, 36.0, 35.6, 31.6; HRMS (ESI) *m*/*z* [M + H]^+^ calcd for C_11_H_13_BrNO 254.0175 and 256.0155, found 254.0175 and 256.0155.

**8**: pale yellow solid; mp 146–147 °C; ^1^H NMR (400 MHz, CDCl_3_, 298 K) δ 8.03 (d, *J* = 7.8 Hz, 1H), 7.47 (td, *J* = 7.5, 1.4 Hz, 1H), 7.36 (t, *J* = 7.5 Hz, 1H), 7.24 (d, *J* = 7.6 Hz, 1H), 6.37 (s, 1H), 4.05 (t, *J* = 6.1 Hz, 1H), 3.59–3.45 (m, 2H), 3.21 (dd, *J* = 15.7, 5.2 Hz, 1H), 2.83 (dd, *J* = 15.7, 7.1 Hz, 1H), 2.18 (ddt, *J* = 13.8, 7.9, 5.8 Hz, 1H), 2.07 (ddt, *J* = 14.2, 8.0, 5.9 Hz, 1H); ^13^C{^1^H} NMR (126 MHz, CDCl_3_, 298 K) δ 166.4, 137.4, 132.5, 128.6, 128.0, 127.7, 127.3, 49.4, 37.6, 33.5, 29.4; HRMS (ESI) *m*/*z* [M + H]^+^ calcd for C_11_H_13_BrNO 254.0175 and 256.0155, found 254.0177 and 256.0153.

#### Synthesis of **7** Using Catalyst **9**

Rh(III) catalyst **9** was prepared according to a literature method.^44^ The regioselective synthesis of **7** using catalyst **9** was based on a modified literature method.^44^ Without any particular precautions to extrude oxygen or moisture, *O*-Piv benzohydoxamate (237.3 mg, 1 mmol, 1 equiv), catalyst **9** (25.0 mg, 0.025 mmol, 0.025 equiv), and cesium acetate (48.0 mg, 0.25 mmol, 0.25 equiv) were added to an 8 mL vial equipped with a stir bar. MeOH (5 mL) was then added followed immediately by 4-bromo-1-butene (270.0 mg, 203 μL, 2 mmol, 2 equiv). The reaction mixture was stirred at room temperature and monitored by TLC and ^1^H NMR. After the reaction had reached completion, the solvent was removed and the residue was purified by column chromatography on silica gel (20% to 67% EtOAc/hexanes) to give compound **7** (202 mg, 79%). **7** was further recrystallized from boiling EtOAc/hexane before the cyclization tests.

#### Synthesis of **10**

AgBF_4_ (hygroscopic, 60.4 mg, 0.31 mmol, 1.5 equiv) was added to an 8 mL vial and dried under high vacuum for 0.5–1 h (covered by aluminum foil). Then, compound **7** (51.4 mg, 0.2 mmol, 1 equiv) was added to the vial under N_2_, followed by the addition of anhydrous CH_3_CN (2 mL). The vial was covered by aluminum foil and placed in a preheated oil bath (80 °C) that was stirred for 4 h until the completion of the reaction was confirmed by ^1^H NMR. The precipitate of AgBr was filtered via a syringe filter (0.2 μm PTFE) and washed with anhydrous CH_3_CN (2 × 0.5 mL). The combined washing solution was concentrated under a rotary evaporator before anhydrous Et_2_O was added. The obtained precipitate or oil after centrifugation was further purified by reprecipitation from anhydrous CH_3_CN/Et_2_O two or three times to give amidium **10** as a white solid (33 mg, 63%): ^1^H NMR (400 MHz, CD_3_CN, 298 K) δ 8.12 (ddt, *J* = 7.9, 1.3, 0.6 Hz, 1H), 7.83 (td, *J* = 7.6, 1.4 Hz, 1H), 7.62–7.52 (m, 2H), 6.57 (br, 1H), 4.09 (ddd, *J* = 13.1, 11.0, 4.3 Hz, 1H), 4.00 (d, *J* = 11.2 Hz, 1H), 3.89 (dd, *J* = 6.2, 3.5 Hz, 1H), 3.66–3.53 (m, 2H), 2.68 (ddt, *J* = 12.8, 10.9, 6.4 Hz, 1H), 2.10–2.00 (m, 1H); ^13^C{^1^H} NMR (101 MHz, CD_3_CN, 298 K) δ 167.6, 149.0, 139.1, 132.6, 130.1, 128.5, 122.2, 60.6, 51.5, 40.4, 30.7.

The crystal of amidium **10** was obtained using the vapor diffusion method under an anhydrous atmosphere as reported previously.^15^**10** was dissolved in 0.5 mL of dry DCM in a 4 mL vial (any insoluble solid was removed by passing the sample through a PTFE syringe filter). The 4 mL vial was placed inside a 20 mL vial containing 5 mL of dry Et_2_O. The 20 mL vial was capped and placed inside a sealed glass jar containing P_2_O_5_ as the desiccant until crystals formed.

#### *In Situ* Formation of **2** from **10**

To an NMR sample solution of **10** in CD_3_CN (dried by 4 Å molecular sieves) was added cesium carbonate (2 equiv). After 10 min, the sample was filtered through a piece of cotton. NMR spectra of the filtrate confirmed the clean formation of twisted amide **2**: ^1^H NMR (400 MHz, CD_3_CN, 298 K) δ 7.90 (dd, *J* = 8.0, 1.0 Hz, 1H), 7.55 (td, *J* = 7.5, 1.3 Hz, 1H), 7.40–7.31 (m, 2H), 3.50–3.27 (m, 3H), 3.09 (dddd, *J* = 12.8, 8.7, 4.4, 2.0 Hz, 1H), 2.87 (dd, *J* = 11.6, 3.4 Hz, 1H), 2.38 (dtd, *J* = 16.8, 6.8, 4.4 Hz, 1H), 1.75 (dddd, *J* = 12.6, 8.1, 6.1, 1.4 Hz, 1H); ^13^C{^1^H} NMR (101 MHz, CD_3_CN, 298 K) δ 182.3, 151.9, 134.6, 130.4, 129.2, 128.2, 126.7, 59.9, 51.5, 39.8, 32.3.

## Data Availability

The data underlying this study are available in the published article and its Supporting Information.

## Supporting Information Available

Additional experimental data and figures, computational data for the optimized ground state geometry of **2**, and NMR spectra (PDF)
