# Supplementary material for: Halide Abstraction-Mediated Synthesis of a Highly Twisted Amide
Source: J Org Chem. 2024 Aug 16;89(17):12779–84. doi: 10.1021/acs.joc.4c01192 (PMC11382264; doi:10.1021/acs.joc.4c01192)
Supplement: Supplementary file 1 — jo4c01192_si_001.pdf [file jo4c01192_si_001.pdf]

Supporting Information

Halide Abstraction-Mediated Synthesis of a Highly Twisted Amide

Mizhi Xu,[a] Krista K. Bullard,[a] John Bacsa,[c] Will R. Gutekunst*[a,b]

[a]School of Chemistry and Biochemistry, [b]School of Materials Science and Engineering, Georgia Institute of Technology, 901 Atlantic Drive NW, Atlanta, Georgia 30332, United States

[c]X-ray Crystallography Center, Department of Chemistry, Emory University, 1515 Dickey Drive, Atlanta, Georgia 30322, United States

*Email: will.gute@chemistry.gatech.edu

TABLE OF CONTENTS

ADDITIONAL EXPERIMENTAL DATA **S1**

COMPUTATIONAL STUDIES **S3**

X-RAY CRYSTALLOGRAPHIC DATA **S4**

NMR SPECTRA **S15**

REFERENCE **S21**

ADDITIONAL EXPERIMENTAL DATA

Synthesis of Twisted Amide 2 from Pyrrolidinium 3 Using Cs_2CO_3

Under N_2, to a mixture of pyrrolidinium salt **3** (4.8 mg, 0.02 mmol, 1 equiv) and Cs_2CO_3 (9.8 mg, 0.03 mmol, 1.5 equiv) in an 8 mL vial was added dry acetonitrile (2 mL, 0.01 M for **3**). The reaction mixture was then stirred at room temperature for 30 minutes, before sampled for ^{1}H NMR and GC-MS analysis. Formation of deprotonated pyrrolidine **4** was observed based on ^{1}H NMR and GC-MS (*m/z* = 205.1). Further heating the reaction mixture at 60 °C overnight led to conversion of **4** to twisted **2**, as shown by ^{1}H NMR and GC-MS (*m/z* = 173.1).

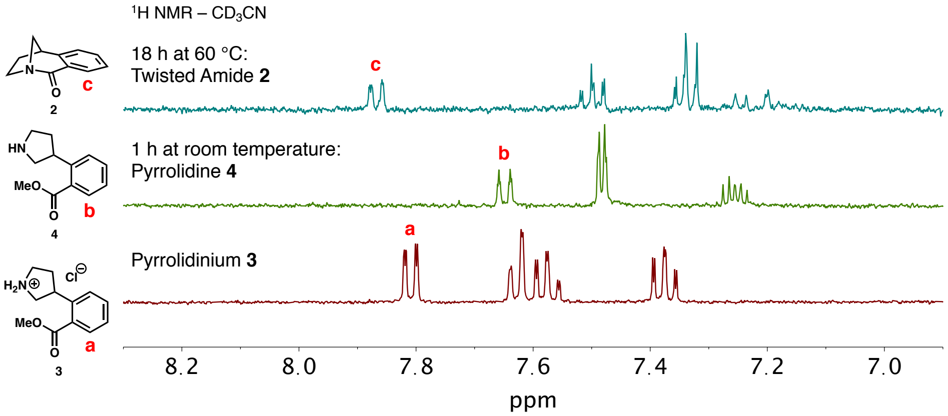

Figure S1. ^{1}H NMR (400 MHz, CD_3CN, 298 K) of pyrrolidium **3**, and formation of deprotonated **4** and twisted amide **2** using Cs_2CO_3.

Synthesis of Twisted Amide 2 from Pyrrolidinium 3 Using DBU

Under N_2, to a solution of pyrrolidinium salt **3** (12.1 mg, 0.05 mmol, 1 equiv) in anhydrous acetonitrile (0.5 mL, 0.1 M for **3**) in an 8 mL vial was added DBU (8.4 mg, 8 µL, 0.055 mmol, 1.1 equiv). The reaction was stirred at room temperature, and aliquots of reaction mixture were taken at different time points for ^1H NMR analysis. As shown by ^1H NMR spectra, at early stage of the reaction, deprotonation of **3** generated **4** that rapidly cyclized to form twisted amide **2**. Further stirring the reaction mixture eventually led to broad peaks likely due to oligomerization of **2**.

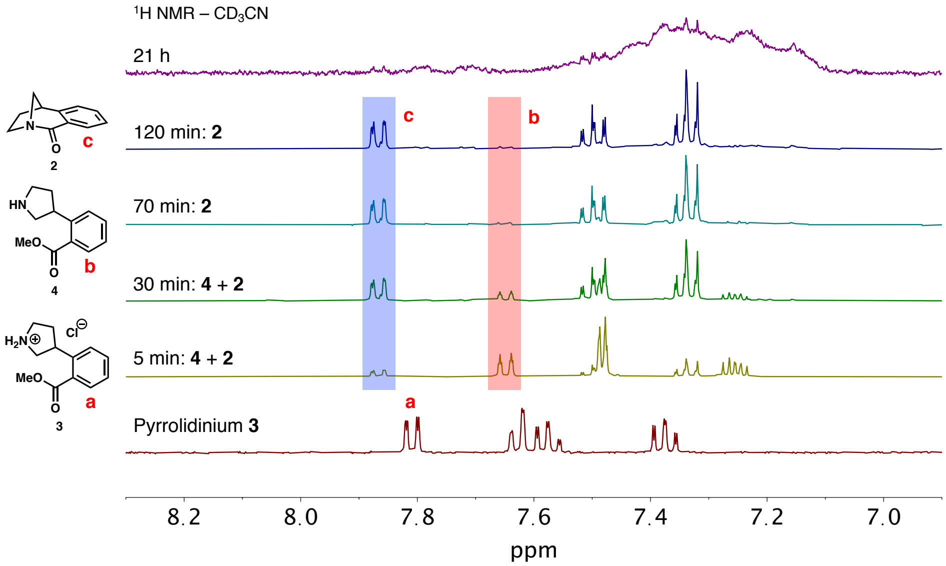

Figure S2. Stacked ^1H NMR spectra (400 MHz, CD_3CN, 298 K) of the reaction between **3** and DBU at different time points.

Attempted Reaction Between 7 and NaH

Under N_2, a solution of 4-(2-bromoethyl)-3,4-dihydroisoquinolone **7** (15.9 mg, 0.063 mmol, 1 equiv) and NaH (6 mg, 60% in mineral oil) in dry THF (1 mL) was stirred at 65 ºC for 6 h. The reaction mixture was analyzed by ^1H NMR.

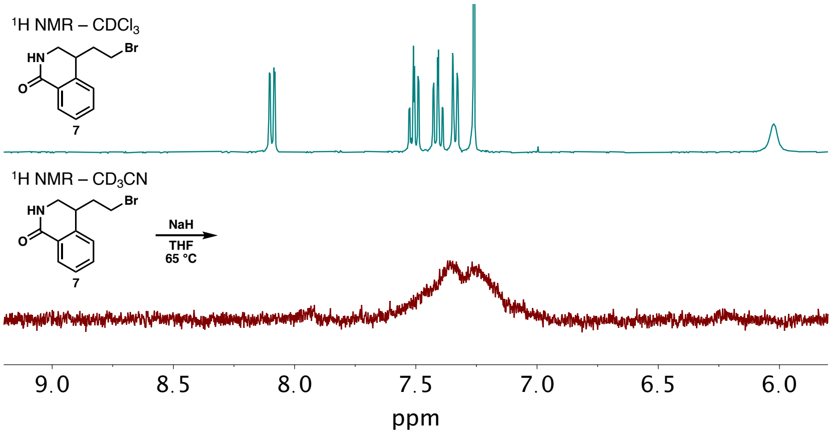

Figure S3. ^1H NMR (400 MHz, CD_3CN, 298 K) of crude reaction between **7** and NaH.

Compare Two Pathways in Synthesis of Twisted Amide 2

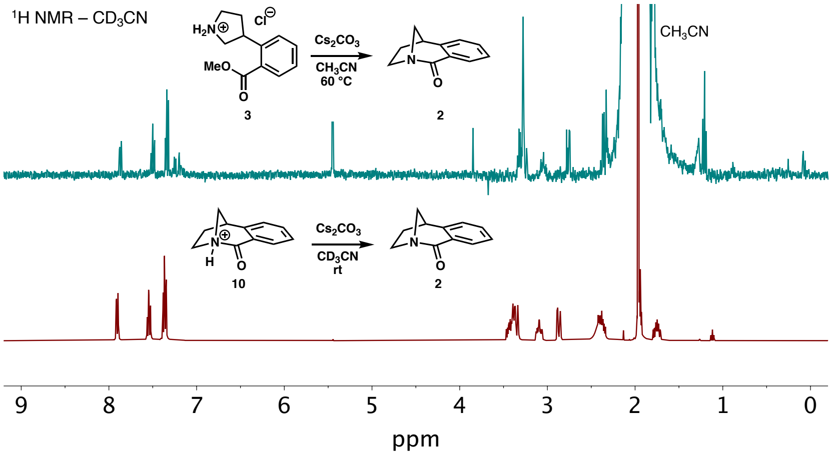

Figure S4. Stacked ^{1}H NMR spectra (400 MHz, CD_3CN, 298 K) of twisted amide **2** synthesized from pyrrolidinium **3** and amidium **10**, respectively.

Synthesis of 11

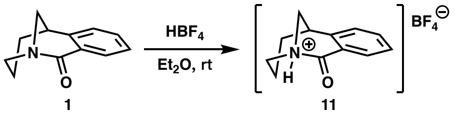

The amidium **11** was prepared according to a literature method.[1] Under N_2, to a solution of twisted amide **1**[2] (93.6 mg, 0.5 mmol, 1 equiv) in anhydrous Et_2O (2.5 mL) in a 20 mL vial was added the HBF_4 solution in Et_2O (50-55% w/w, 73 μL, containing 1 equiv HBF_4), and the reaction mixture was stirred at room temperature. After the complete reaction confirmed by ^{1}H NMR, the solvent was removed by rotary evaporation. The resulting solid was washed with anhydrous diethyl ether twice to afford the amidium salt **11** as a white solid. Melting point: 147-148 °C; ^{1}H NMR (500 MHz, CD_3CN, 298 K) δ = 8.17 (d, *J* = 7.9 Hz, 1H), 7.88 (t, *J* = 7.6 Hz, 1H), 7.60 (t, *J* = 7.7 Hz, 1H), 7.53 (d, *J* = 7.7 Hz, 1H), 4.09 (d, *J* = 12.3 Hz, 1H), 3.78-3.66 (m, 2H), 3.66-3.55 (m, 1H), 3.52 (s, 1H), 2.24-2.10 (m, 1H), 1.80 (d, *J* = 12.6 Hz, 2H), 1.64-1.48 (m, 1H); ^{13}C{^{1}H} NMR (101 MHz, CD_3CN, 298 K) δ = 166.3, 146.0, 139.4, 131.3, 129.9, 129.9, 126.1, 53.8, 53.5, 32.4, 26.6, 16.7.

The crystal of amidium **11** was obtained in similar procedure and setup to that for amidium **10**, except that anhydrous CH_3CN was used to dissolve **11** instead of DCM.

COMPUTATIONAL STUDIES

All the calculations were carried out using Psi4 with the standard grid size (75,302).[3] All of the geometry optimizations and frequency analysis were performed at the B3LYP-D3MBJ/6-311++G(d,p) level of theory in the gas phase. The absence of imaginary frequencies was used to characterize the structures as minima on the potential energy surface. All of the optimized geometries were verified as minima (no imaginary frequencies). Electronic and thermal energies were calculated for all structures. Energetic parameters were calculated under standard conditions (298.15 K and 1 atm).[4–7] Winkler-Dunitz parameters and bond lengths were calculated using Mercury (version: 4.0.0).

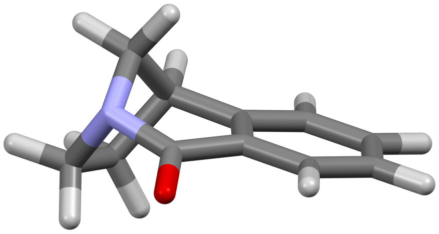

Figure S5. Optimized geometry of twisted amides **2** calculated in the main manuscript.

Table S1. Calculated Winkler-Dunitz Parameters and Bond Lengths of Twisted Amides 1 and 2[a]

Entry	Amide	τ (°)	χ_N (°)	($\tau + \chi_N$) (°)	N-C(O) (Å)	C=O (Å)	C(O)-C(Ar) (Å)
1	1	36.50	50.93	87.43	1.405	1.218	1.501
2	2	51.61	66.38	117.99	1.433	1.211	1.499

[a]Calculated using B3LYPD3MBJ/6-311++G(d,p) level of theory.

Energy and Geometry of Twisted Amide 2

Total energy: -555.90971444 au

Total energy including zero-point energy and thermal corrections: -555.71109228 au

Geometry:

C	0.714978458208	-1.832003003514	-0.973240011239
C	0.144654832385	-0.765947999984	-0.273351480908
C	0.953928216395	0.261561427612	0.251488404625
C	2.332491195319	0.202376649833	0.050031848200
C	2.093109472862	-1.889165581497	-1.149777360826
C	2.898715517558	-0.868036799453	-0.642141571890
H	3.973442299193	-0.907972062102	-0.782006670294
H	2.539650924823	-2.717480619878	-1.687619597255
C	0.271826551564	1.356440826704	1.046995307542
C	-0.558634032895	2.335442796136	0.152672336770
C	-1.977881993462	1.678527780111	0.090366060304
N	-1.822255182317	0.353289075341	0.755379236936
C	-1.343309672758	-0.606263039741	-0.195089913329
C	-0.847697152621	0.644576897836	1.823615141946
H	-0.526361903155	-0.268407005916	2.325423876778
H	-1.330657885841	1.294039926362	2.559381479856
H	0.993720452612	1.885736783258	1.671687189326
O	-2.099928970099	-1.154926742569	-0.965427920561
H	0.063735264158	-2.595929335869	-1.381407372814
H	2.967957179549	0.987466429795	0.446739139696
H	-2.707598807881	2.263519683331	0.653833962793
H	-2.360502893281	1.541755552078	-0.919820861427
H	-0.113893406737	2.451730357864	-0.836681088664
H	-0.602974513868	3.324441280639	0.615099797649

The optimized energy and geometry of twisted amide **1** has been reported before.[8]

X-RAY CRYSTALLOGRAPHIC DATA

X-Ray Crystallographic Structure of 10

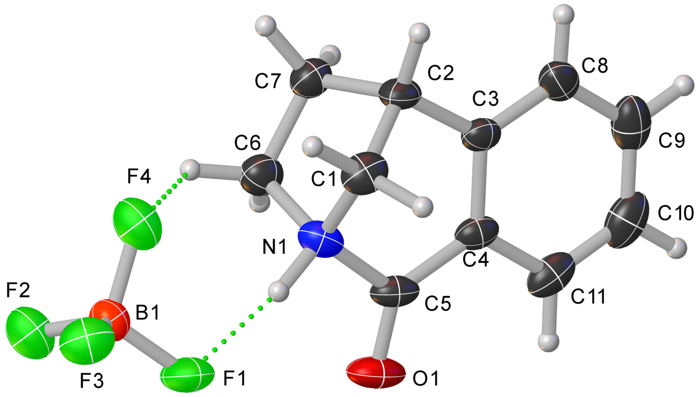

Single colorless needle-shaped crystals of **10** were chosen from the sample as supplied (in ether). A suitable crystal with dimensions 0.22 × 0.20 × 0.12 mm^3 was selected and mounted on a loop with paratone on a XtaLAB Synergy, Dualflex, HyPix diffractometer. The crystal was kept at a steady T = 173.00(10) K during data collection. The structure was solved with the **ShelXT** 2018/2 (Sheldrick, 2018) solution program using dual-space methods and by using **Olex2** 1.3-alpha (Dolomanov et

al., 2009) as the graphical interface. The model was refined with **ShelXL** 2018/3 (Sheldrick, 2015) using full matrix least squares minimisation on $\boldsymbol{F}^2$.

Table S2. Crystal Data for 10.

Formula	$C_{11}H_{12}BF_4NO$
$D_{calc.}$/ g cm^{-3}	1.483
μ/mm^{-1}	1.200
Formula Weight	261.03
Color	colorless
Shape	needle-shaped
Size/mm^3	0.22×0.20×0.12
T/K	173.00(10)
Crystal System	monoclinic
Space Group	$P2_1/c$
a/Å	6.9341(3)
b/Å	16.2426(7)
c/Å	10.3887(4)
α/°	90
β/°	91.889(4)
γ/°	90
V/Å^3	1169.42(8)
Z	4
Z'	1
Wavelength/Å	1.54184
Radiation type	Cu K_α
Θ_{min}/°	5.056
Θ_{max}/°	65.052
Measured Refl's.	13963
Indep't Refl's	1975
Refl's I≥2 σ(I)	1727
R_{int}	0.0411
Parameters	194
Restraints	67
Largest Peak	0.513
Deepest Hole	-0.499
GooF	1.043
wR_2 (all data)	0.2546
wR_2	0.2465
R_1 (all data)	0.0940
R_1	0.0869

Table S3. Fractional Atomic Coordinates (×10^4) and Equivalent Isotropic Displacement Parameters (Å^2×10^3) for 10. U_{eq} is defined as 1/3 of the trace of the orthogonalised U_{ij}.

Atom	x	y	z	U_{eq}
O1	4033(3)	5853.8(18)	7488(3)	77.8(9)
N1	6439(4)	6349.4(19)	6233(3)	58.2(8)
C7	9633(5)	5821(2)	6122(4)	59.4(10)
C1	7805(5)	7053(2)	6368(4)	61.8(10)
C9	9730(8)	6048(3)	10578(4)	78.4(13)

Atom	x	y	z	U_{eq}
C10	7831(8)	5847(2)	10775(4)	77.2(13)
C6	7581(5)	5659(3)	5662(4)	65.6(10)
C4	7080(4)	6101.7(19)	8552(3)	48.9(8)
C3	9006(4)	6324(2)	8337(3)	48.5(8)
C5	5669(4)	6079(2)	7501(4)	55.2(9)
C11	6510(6)	5875(2)	9787(4)	64.5(11)
C2	9539(5)	6573(2)	6998(3)	56.3(9)
C8	10315(5)	6296(2)	9362(4)	63.4(10)
B1_1	6558(8)	3256(3)	6839(6)	58.8(16)
F1_1	5484(11)	2812(4)	5910(6)	85.4(10)
F2_1	8505(8)	3290(4)	6567(8)	80.3(17)
F3_1	5821(11)	4049(3)	6907(8)	85.4(10)
F4_1	6359(11)	2870(5)	8024(6)	85.4(10)
B1_2	4141(7)	6631(3)	3239(4)	58.8(16)
F1_2	3321(7)	6579(5)	4449(5)	91(2)
F2_2	3596(11)	7341(3)	2575(6)	80.3(17)
F3_2	3711(9)	5930(3)	2520(6)	85.4(10)
F4_2	6130(7)	6680(4)	3452(5)	85.4(10)
B1_3	13561(11)	6704(5)	3442(8)	58.8(16)
F1_3	13750(16)	7114(6)	4617(9)	85.4(10)
F2_3	13810(20)	7216(8)	2388(11)	85.4(10)
F3_3	14882(13)	6060(6)	3413(10)	85.4(10)
F4_3	11698(12)	6385(6)	3359(11)	85.4(10)

Table S4. Anisotropic Displacement Parameters (×10⁴) for 10. The anisotropic displacement factor exponent takes the form: $-2\pi^2 [h^2a^{*2} \times U_{11} + ... + 2hka^* \times b^* \times U_{12}]$.

Atom	U_{11}	U_{22}	U_{33}	U_{23}	U_{13}	U_{12}
O1	36.0(13)	70.4(18)	128(3)	-1.6(16)	12.8(14)	-7.1(11)
N1	43.8(15)	57.0(17)	73.3(19)	4.3(14)	-5.0(13)	0.3(12)
C7	58(2)	59(2)	63(2)	-3.7(16)	10.8(16)	2.4(16)
C1	68(2)	44.5(19)	74(2)	6.5(16)	15.0(18)	-3.5(15)
C9	105(4)	67(3)	62(2)	-11.4(19)	-12(2)	31(2)
C10	119(4)	50(2)	65(2)	0.9(17)	26(3)	16(2)
C6	60(2)	63(2)	74(2)	-8.0(18)	5.1(18)	3.6(17)
C4	43.9(17)	36.6(16)	67(2)	0.1(13)	15.4(15)	3.4(12)
C3	40.2(16)	46.3(17)	59.6(19)	-7.3(14)	8.0(14)	4.1(12)
C5	36.4(16)	44.2(17)	86(2)	2.7(16)	12.8(15)	-1.0(13)
C11	79(3)	37.9(18)	78(3)	-0.9(16)	34(2)	1.2(16)
C2	36.4(16)	66(2)	67(2)	-6.7(16)	9.7(14)	-11.5(14)
C8	54(2)	67(2)	69(2)	-17.5(18)	-4.2(17)	14.4(17)
B1_1	61(2)	57(2)	59(2)	2.9(16)	-4.3(18)	0.8(17)
F1_1	87(2)	83.3(17)	84(2)	4.4(16)	-17.2(15)	-2.5(14)
F2_1	80(3)	63(3)	99(4)	14(3)	15(3)	0(2)
F3_1	87(2)	83.3(17)	84(2)	4.4(16)	-17.2(15)	-2.5(14)
F4_1	87(2)	83.3(17)	84(2)	4.4(16)	-17.2(15)	-2.5(14)
B1_2	61(2)	57(2)	59(2)	2.9(16)	-4.3(18)	0.8(17)
F1_2	44(3)	137(6)	92(4)	4(4)	13(3)	1(3)
F2_2	80(3)	63(3)	99(4)	14(3)	15(3)	0(2)

Atom	U_{11}	U_{22}	U_{33}	U_{23}	U_{13}	U_{12}
F3_2	87(2)	83.3(17)	84(2)	4.4(16)	-17.2(15)	-2.5(14)
F4_2	87(2)	83.3(17)	84(2)	4.4(16)	-17.2(15)	-2.5(14)
B1_3	61(2)	57(2)	59(2)	2.9(16)	-4.3(18)	0.8(17)
F1_3	87(2)	83.3(17)	84(2)	4.4(16)	-17.2(15)	-2.5(14)
F2_3	87(2)	83.3(17)	84(2)	4.4(16)	-17.2(15)	-2.5(14)
F3_3	87(2)	83.3(17)	84(2)	4.4(16)	-17.2(15)	-2.5(14)
F4_3	87(2)	83.3(17)	84(2)	4.4(16)	-17.2(15)	-2.5(14)

Table S5. Bond Lengths in Å for 10.

Atom	Atom	Length/Å
O1	C5	1.192(4)
N1	C1	1.488(5)
N1	C6	1.505(5)
N1	C5	1.503(5)
C7	C6	1.509(5)
C7	C2	1.526(5)
C1	C2	1.559(5)
C9	C10	1.378(7)
C9	C8	1.398(6)
C10	C11	1.354(7)
C4	C3	1.408(4)
C4	C5	1.442(5)
C4	C11	1.404(5)
C3	C2	1.507(5)

Atom	Atom	Length/Å
C3	C8	1.378(5)
B1_1	F1_1	1.3993(19)
B1_1	F2_1	1.390(4)
B1_1	F3_1	1.388(4)
B1_1	F4_1	1.392(4)
B1_2	F1_2	1.399(3)
B1_2	F2_2	1.389(3)
B1_2	F3_2	1.389(3)
B1_2	F4_2	1.392(3)
B1_3	F1_3	1.393(2)
B1_3	F2_3	1.391(2)
B1_3	F3_3	1.391(2)
B1_3	F4_3	1.391(2)

Table S6. Bond Angles in ° for 10.

Atom	Atom	Atom	Angle/°
C1	N1	C6	105.5(3)
C1	N1	C5	112.7(3)
C5	N1	C6	109.5(3)
C6	C7	C2	105.7(3)
N1	C1	C2	97.8(3)
C10	C9	C8	120.6(4)
C11	C10	C9	120.4(4)
N1	C6	C7	104.4(3)
C3	C4	C5	120.6(3)
C11	C4	C3	120.6(4)
C11	C4	C5	118.7(3)
C4	C3	C2	118.6(3)
C8	C3	C4	118.2(3)
C8	C3	C2	123.2(3)
O1	C5	N1	116.4(3)
O1	C5	C4	129.7(4)
C4	C5	N1	113.8(3)
C10	C11	C4	119.8(4)
C7	C2	C1	101.4(3)
C3	C2	C7	110.6(3)

Atom	Atom	Atom	Angle/°
C3	C2	C1	108.2(3)
C3	C8	C9	120.3(4)
F2_1	B1_1	F1_1	112.3(5)
F2_1	B1_1	F4_1	108.8(3)
F3_1	B1_1	F1_1	109.0(3)
F3_1	B1_1	F2_1	109.6(3)
F3_1	B1_1	F4_1	109.0(3)
F4_1	B1_1	F1_1	108.2(3)
F2_2	B1_2	F1_2	112.7(4)
F2_2	B1_2	F4_2	106.5(4)
F3_2	B1_2	F1_2	110.3(4)
F3_2	B1_2	F2_2	111.3(4)
F3_2	B1_2	F4_2	109.0(4)
F4_2	B1_2	F1_2	106.9(4)
F2_3	B1_3	F1_3	113.1(5)
F2_3	B1_3	F3_3	109.5(5)
F2_3	B1_3	F4_3	108.2(5)
F3_3	B1_3	F1_3	109.6(5)
F4_3	B1_3	F1_3	107.0(4)
F4_3	B1_3	F3_3	109.3(5)

Table S7. Torsion Angles in ° for 10.

Atom	Atom	Atom	Atom	Angle/°
N1	C1	C2	C7	47.3(3)
N1	C1	C2	C3	-69.0(3)
C1	N1	C6	C7	28.5(4)
C1	N1	C5	O1	143.9(3)
C1	N1	C5	C4	-38.5(4)
C9	C10	C11	C4	0.6(6)
C10	C9	C8	C3	-1.6(6)
C6	N1	C1	C2	-47.0(3)
C6	N1	C5	O1	-99.0(4)
C6	N1	C5	C4	78.6(3)
C6	C7	C2	C1	-31.4(4)
C6	C7	C2	C3	83.2(3)
C4	C3	C2	C7	-74.4(4)
C4	C3	C2	C1	35.8(4)
C4	C3	C8	C9	0.3(5)
C3	C4	C5	O1	174.3(4)
C3	C4	C5	N1	-2.9(4)
C3	C4	C11	C10	-1.9(5)
C5	N1	C1	C2	72.5(3)
C5	N1	C6	C7	-93.1(3)
C5	C4	C3	C2	2.6(5)
C5	C4	C3	C8	-176.8(3)
C5	C4	C11	C10	176.3(3)
C11	C4	C3	C2	-179.2(3)
C11	C4	C3	C8	1.4(5)
C11	C4	C5	O1	-3.9(5)
C11	C4	C5	N1	178.9(3)
C2	C7	C6	N1	3.0(4)
C2	C3	C8	C9	-179.1(3)
C8	C9	C10	C11	1.1(6)
C8	C3	C2	C7	105.0(4)
C8	C3	C2	C1	-144.8(3)

Table S8. Hydrogen Fractional Atomic Coordinates (×10^4) and Equivalent Isotropic Displacement Parameters (Å^2×10^3) for 10. U_{eq} is defined as 1/3 of the trace of the orthogonalised U_{ij}.

Atom	x	y	z	U_{eq}
H1A	13800(3)	6928(2)	8073(3)	36.1(10)
H1B	12540(4)	7158(2)	8731(3)	35.0(10)
H2A	11405(3)	6640(2)	7207(3)	32.2(9)
H2B	12375(4)	7408(2)	6695(3)	35.1(10)
H3A	12185(4)	5993(2)	5550(3)	35.4(10)
H3B	13557(3)	6219(2)	6014(3)	35.1(10)
H4	13236(3)	4636(2)	6241(3)	32.1(9)
H5A	13628(3)	4529(2)	8249(3)	33.5(10)
H5B	14342(3)	5459(2)	7710(3)	34.9(10)

Atom	x	y	z	U_{eq}
H9	11412(3)	4006(2)	5582(3)	32.8(10)
H11	9044(4)	3443(3)	8046(3)	38.5(11)
H12	10100(3)	4432(2)	9313(3)	35.4(10)
H13D	10674(4)	2702(3)	4987(3)	42.6(11)
H13E	9786(4)	3574(3)	4578(3)	45.1(12)
H13F	9237(4)	2470(3)	4711(3)	49.5(12)
H1BA	7039(4)	7726(3)	6379(3)	42.9(11)
H1BB	8299(4)	7735(3)	7174(3)	46.8(11)
H2BA	6042(3)	6919(2)	7759(3)	38.1(10)
H2BB	6713(4)	7814(3)	8418(3)	43.7(11)
H3BA	6862(4)	6310(3)	9418(3)	42.2(11)
H3BB	8144(4)	6845(3)	9209(3)	44.2(11)
H4B	8424(3)	5297(2)	8814(3)	32.2(10)
H5BA	8989(4)	5416(3)	6877(3)	38.9(10)
H5BB	9276(3)	6444(2)	7563(3)	38.4(10)
H9B	6853(4)	4274(2)	9201(3)	33.5(10)
H11B	4919(3)	3372(2)	6421(3)	33.4(10)
H12B	5687(4)	4629(2)	5357(3)	34.0(10)
H13A	6353(4)	2853(3)	9751(3)	46.0(12)
H13B	5265(4)	3623(3)	9954(3)	44.1(12)
H13C	4953(4)	2467(3)	9805(3)	52.6(13)

Table S9. Hydrogen Bond Information for 10.

D	H	A	d(D-H)/Å	d(H-A)/Å	d(D-A)/Å	D-H-A/deg
C7	H7A	F4_3	1.05	2.35	3.376(12)	164.1
C6	H6A	F4_2	1.05	2.24	2.980(7)	125.9

Table S10. Atomic Occupancies for all atoms that are not fully occupied in 10.

Atom	Occupancy	Atom	Occupancy	Atom	Occupancy	Atom	Occupancy
B1_1	0.328(3)	F4_1	0.328(3)	F3_2	0.426(2)	F2_3	0.246(3)
F1_1	0.328(3)	B1_2	0.426(2)	F4_2	0.426(2)	F3_3	0.246(3)
F2_1	0.328(3)	F1_2	0.426(2)	B1_3	0.246(3)	F4_3	0.246(3)
F3_1	0.328(3)	F2_2	0.426(2)	F1_3	0.246(3)		

X-Ray Crystallographic Structure of 11

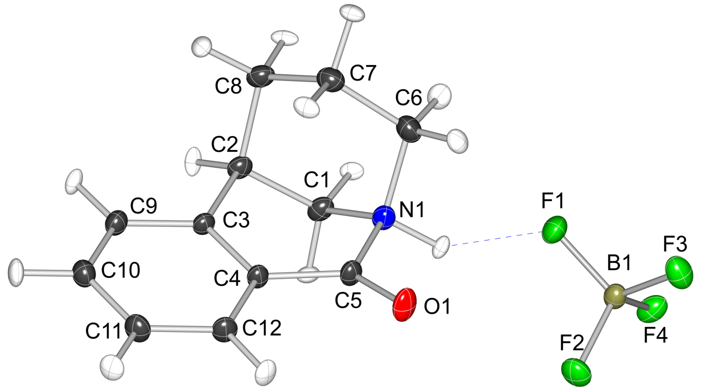

Single colorless prism-shaped crystals of **11** crystallized from DCM and ether by vapor diffusion. A suitable crystal with dimensions 0.27 × 0.23 × 0.15 mm3 was selected and mounted on a loop with paratone on a XtaLAB Synergy-S diffractometer.

The crystal was kept at a steady T = 100(1) K during data collection. The structure was solved with the **ShelXT** 2018/2 (Sheldrick, 2018) solution program using dual methods and by using **Olex2** 1.3-alpha (Dolomanov et al., 2009) as the graphical interface. The model was refined with **olex2.refine** 1.3-alpha (Bourhis et al., 2015) using full matrix least squares minimisation on F^2.

Table S11. Crystal Data for 11.

Formula	$C_{12}H_{14}BF_4NO$
$D_{calc.}$/ g cm^{-3}	1.504
μ/mm^{-1}	1.185
Formula Weight	275.069
Color	colorless
Shape	prism-shaped
Size/mm^3	0.27×0.23×0.15
T/K	100.00(10)
Crystal System	triclinic
Space Group	P-1
a/Å	7.4205(2)
b/Å	8.6707(3)
c/Å	9.7368(3)
α/°	96.945(2)
β/°	94.256(2)
γ/°	101.078(3)
V/Å^3	607.23(3)
Z	2
Z'	1
Wavelength/Å	1.54184
Radiation type	Cu K_α
Θ_{min}/°	4.60
Θ_{max}/°	68.22
Measured Refl's.	8240
Indep't Refl's	2220
Refl's I≥2 σ(I)	2097
R_{int}	0.0447
Parameters	299
Restraints	261
Largest Peak	0.2582
Deepest Hole	-0.1951
GooF	1.1636
wR_2 (all data)	0.0669
wR_2	0.0650
R_1 (all data)	0.0286
R_1	0.0265

Table S12. Fractional Atomic Coordinates (×10^4) and Equivalent Isotropic Displacement Parameters (Å^2×10^3) for 11. U_{eq} is defined as 1/3 of the trace of the orthogonalised U_{ij}.

Atom	x	y	z	U_{eq}
F4	6460.4(7)	944.2(7)	3058.0(5)	20.90(15)
F2	7125.3(8)	669.4(7)	5322.6(5)	23.22(16)
F3	8548.2(7)	2858.9(7)	4452.0(5)	23.60(16)
F1	5502.9(8)	2543.5(7)	4774.5(5)	22.29(15)

Atom	x	y	z	U_{eq}
O1	5831.0(9)	2335.1(9)	8489.5(6)	22.17(18)
N1	3379.1(10)	2559.5(9)	6977.4(7)	16.48(19)
C4	2902.8(12)	1964.0(11)	9411.2(8)	13.86(19)
C12	3589.1(12)	1609.4(11)	10692.2(9)	16.8(2)
C3	1074.6(12)	2160.8(10)	9182.9(8)	13.43(19)
C9	-24.6(12)	2059.5(11)	10284.5(9)	16.3(2)
C8	583.0(13)	4333.4(12)	7789.2(9)	18.3(2)
C5	4190.7(12)	2238.5(11)	8357.4(8)	15.7(2)
C1	1362.9(12)	1827.3(11)	6660.4(8)	16.4(2)
C2	342.6(12)	2528.9(11)	7797.1(8)	15.1(2)
C10	673.3(13)	1755.3(11)	11566.7(9)	18.5(2)
C7	2625.0(13)	5136.9(11)	7978.4(9)	19.3(2)
C11	2475.3(13)	1504.2(12)	11772.2(9)	18.9(2)
C6	3704.9(14)	4339.6(12)	6935.5(9)	20.8(2)
B1	6909.8(14)	1747.8(13)	4409.0(9)	15.7(2)

Table S13. Anisotropic Displacement Parameters (×10[4]) for 11. The anisotropic displacement factor exponent takes the form: $-2\pi^2 [h^2a^{*2} \times U_{11} + \ldots + 2hka^* \times b^* \times U_{12}]$.

Atom	U_{11}	U_{22}	U_{33}	U_{23}	U_{13}	U_{12}
F4	22.2(3)	27.2(3)	15.2(2)	12.4(2)	-1.6(2)	1.1(2)
F2	25.3(3)	27.4(3)	19.6(3)	7.2(2)	0.7(2)	11.7(2)
F3	17.0(3)	31.1(3)	22.7(3)	1.5(2)	-0.2(2)	10.6(2)
F1	22.1(3)	25.3(3)	23.3(3)	10.9(2)	8.6(2)	4.8(2)
O1	10.3(3)	37.5(4)	20.8(3)	7.8(3)	1.9(2)	7.6(3)
N1	15.5(4)	21.8(4)	14.9(3)	7.7(3)	2.7(3)	6.3(3)
C4	10.5(4)	18.9(4)	13.7(4)	5.2(3)	1.0(3)	5.1(3)
C12	13.0(4)	22.9(5)	16.4(4)	6.7(3)	-0.9(3)	6.4(3)
C3	10.6(4)	16.2(4)	14.9(4)	5.2(3)	0.9(3)	3.8(3)
C9	13.4(4)	19.9(4)	17.4(4)	5.9(3)	3.5(3)	4.0(3)
C8	19.7(5)	19.2(5)	18.4(4)	9.5(4)	0.6(3)	4.6(3)
C5	10.4(4)	22.1(5)	16.3(4)	5.8(3)	1.8(3)	5.7(3)
C1	15.7(4)	21.3(5)	13.7(4)	8.5(4)	-0.9(3)	2.5(3)
C2	12.8(4)	18.5(5)	15.3(4)	6.5(3)	-0.6(3)	3.3(3)
C10	18.6(5)	21.6(5)	17.0(4)	4.7(4)	4.6(3)	5.8(3)
C7	22.3(5)	18.3(5)	18.5(4)	5.7(4)	1.9(3)	5.6(3)
C11	19.5(5)	24.4(5)	14.3(4)	5.3(4)	0.9(3)	7.3(3)
C6	21.3(5)	22.3(5)	21.1(4)	5.3(4)	5.2(4)	9.6(3)
B1	14.6(5)	21.1(5)	13.0(4)	6.2(4)	1.8(3)	4.8(4)
H8a	45(8)	20(7)	22(3)	13(5)	-7(2)	4(2)
H2	13(2)	51(8)	18(6)	0.1(15)	-0.4(15)	1(4)
H6a	46(8)	62(9)	23(3)	26(6)	5(2)	16(2)
H1a	36(7)	28(7)	15(3)	14(5)	-4(2)	3.2(18)
H12	19(3)	68(10)	36(7)	22(2)	2(3)	21(6)
H1b	28(7)	22(2)	42(7)	10.6(13)	4(5)	6.3(13)
H8b	46(7)	27(7)	34(5)	14(4)	19(3)	4(3)
H7a	43(7)	31(7)	22(3)	8(5)	-5.1(18)	8(2)
H9	17(2)	58(10)	25(6)	18(2)	5(2)	11(6)
H11	40(8)	58(10)	21(4)	18(6)	-1(3)	18(3)

Atom	U_{11}	U_{22}	U_{33}	U_{23}	U_{13}	U_{12}
H6b	22(2)	41(8)	43(7)	2.4(13)	2.7(14)	18(5)
H1	21(6)	37(8)	23(5)	12(4)	6(2)	1(3)
H10	35(6)	57(11)	29(4)	13(5)	19(2)	15(4)
H7b	42(8)	21(2)	44(7)	8.7(18)	6(5)	12.9(15)

Table S14. Bond Lengths in Å for 11.

Atom	Atom	Length/Å
F4	B1	1.3968(11)
F2	B1	1.3888(11)
F3	B1	1.3930(12)
F1	B1	1.4018(12)
O1	C5	1.2000(11)
N1	C5	1.5101(10)
N1	C1	1.5019(11)
N1	C6	1.5217(12)
C4	C12	1.4029(12)
C4	C3	1.4044(12)

Atom	Atom	Length/Å
C4	C5	1.4629(12)
C12	C11	1.3858(13)
C3	C9	1.3960(13)
C3	C2	1.5098(11)
C9	C10	1.3901(13)
C8	C2	1.5413(13)
C8	C7	1.5288(13)
C1	C2	1.5188(12)
C10	C11	1.3993(13)
C7	C6	1.5216(13)

Table S15. Bond Angles in ° for 11.

Atom	Atom	Atom	Angle/°
C1	N1	C5	113.10(7)
C6	N1	C5	109.82(7)
C6	N1	C1	110.50(7)
C3	C4	C12	121.09(8)
C5	C4	C12	117.79(8)
C5	C4	C3	120.97(7)
C11	C12	C4	119.88(8)
C9	C3	C4	118.22(8)
C2	C3	C4	120.74(8)
C2	C3	C9	121.00(8)
C10	C9	C3	120.64(8)
C7	C8	C2	110.98(7)
N1	C5	O1	116.25(8)
C4	C5	O1	127.57(7)
C4	C5	N1	116.00(7)

Atom	Atom	Atom	Angle/°
C2	C1	N1	107.89(7)
C8	C2	C3	111.12(7)
C1	C2	C3	109.16(7)
C1	C2	C8	109.75(7)
C11	C10	C9	120.83(8)
C6	C7	C8	111.27(8)
C10	C11	C12	119.27(8)
C7	C6	N1	111.02(7)
F2	B1	F4	109.47(8)
F3	B1	F4	108.91(7)
F3	B1	F2	109.95(7)
F1	B1	F4	109.38(7)
F1	B1	F2	110.24(7)
F1	B1	F3	108.87(8)

Table S16. Torsion Angles in ° for 11.

Atom	Atom	Atom	Atom	Angle/°
O1	C5	N1	C1	157.48(8)
O1	C5	N1	C6	-78.58(9)
O1	C5	C4	C12	-7.08(12)
O1	C5	C4	C3	168.47(10)
N1	C5	C4	C12	177.93(8)
N1	C5	C4	C3	-6.52(9)
N1	C1	C2	C3	-59.76(8)
N1	C1	C2	C8	62.25(7)

Atom	Atom	Atom	Atom	Angle/°
N1	C6	C7	C8	-52.74(7)
C4	C12	C11	C10	-0.01(11)
C4	C3	C9	C10	-0.66(10)
C4	C3	C2	C8	-92.46(9)
C4	C3	C2	C1	28.72(9)
C12	C11	C10	C9	1.96(11)
C3	C9	C10	C11	-1.63(11)
C3	C2	C8	C7	63.35(8)

Table S17. Hydrogen Fractional Atomic Coordinates (×10⁴) and Equivalent Isotropic Displacement Parameters (Å²×10³) for 11. U_{eq} is defined as 1/3 of the trace of the orthogonalised U_{ij}.

Atom	x	y	z	U_{eq}
H8a	-16(18)	4519(15)	6766(12)	28(3)
H2	-1126(16)	1996(17)	7584(11)	29(3)
H6a	3250(20)	4466(19)	5877(13)	41(4)
H1a	947(17)	2081(15)	5634(11)	26(3)
H12	4958(18)	1390(20)	10843(13)	38(4)
H1b	1221(17)	536(16)	6638(13)	30(3)
H8b	-140(20)	4843(17)	8636(13)	34(3)
H7a	3218(19)	5154(16)	9030(12)	32(3)
H9	-1459(17)	2212(18)	10106(12)	32(3)
H11	2990(19)	1252(19)	12755(13)	38(4)
H6b	5223(18)	4770(18)	7133(14)	35(3)
H1	4140(20)	2088(19)	6210(14)	26(3)
H10	-193(19)	1671(19)	12414(13)	39(4)
H7b	2810(20)	6412(17)	7822(14)	35(3)

Table S18. Hydrogen Bond Information for 11.

**D**	**H**	**A**	d(D-H)/Å	d(H-A)/Å	d(D-A)/Å	D-H-A/deg
N1	H1	F1	1.059(15)	1.822(15)	2.7546(9)	144.5(13)

Table S19. Selected Bond Lengths in Å for 11.

Atom	Atom	Length/Å
N1	H1	1.059(15)
C12	H12	1.072(12)
C9	H9	1.101(12)
C8	H8a	1.103(11)
C8	H8b	1.111(13)
C1	H1a	1.084(11)
C1	H1b	1.101(13)

Atom	Atom	Length/Å
C2	H2	1.093(12)
C10	H10	1.084(13)
C7	H7a	1.082(11)
C7	H7b	1.118(14)
C11	H11	1.067(11)
C6	H6a	1.085(12)
C6	H6b	1.111(13)

Table S20. Selected Bond Angles in ° for 11.

Atom	Atom	Atom	Angle/°
H1	N1	C5	106.5(7)
H1	N1	C1	110.1(8)

Atom	Atom	Atom	Angle/°
H1	N1	C6	106.6(9)
H12	C12	C4	120.9(7)

Atom	Atom	Atom	Angle/°
H12	C12	C11	119.2(7)
H9	C9	C3	118.3(6)
H9	C9	C10	121.1(6)
H8a	C8	C2	107.4(7)
H8a	C8	C7	108.5(7)
H8b	C8	C2	109.4(7)
H8b	C8	C7	109.8(7)
H8b	C8	H8a	110.7(10)
H1a	C1	N1	106.8(7)
H1a	C1	C2	113.5(7)
H1b	C1	N1	107.3(7)
H1b	C1	C2	111.7(7)
H1b	C1	H1a	109.4(9)
H2	C2	C3	109.8(6)
H2	C2	C8	108.4(8)

Atom	Atom	Atom	Angle/°
H2	C2	C1	108.6(7)
H10	C10	C9	120.1(7)
H10	C10	C11	119.0(7)
H7a	C7	C8	111.1(7)
H7a	C7	C6	110.7(8)
H7b	C7	C8	110.8(7)
H7b	C7	C6	107.6(7)
H7b	C7	H7a	105.2(10)
H11	C11	C12	120.5(8)
H11	C11	C10	120.3(8)
H6a	C6	N1	105.3(9)
H6a	C6	C7	111.5(8)
H6b	C6	N1	105.6(8)
H6b	C6	C7	113.6(8)
H6b	C6	H6a	109.4(11)

Table S21. Calculated Winkler-Dunitz Parameters and Bond Lengths of Amidiums 10 and 11[a]

Entry	Amidium	τ (°)	χ_N (°)	($\tau + \chi_N$) (°)	N-C(O) (Å)	C=O (Å)	C(O)-C(Ar) (Å)
1	10	68.72	62.89	131.61	1.503	1.192	1.442
2	11	52.77	56.06	108.83	1.510	1.200	1.463

[a]Calculated based on crystal structures of **10** and **11**.

NMR SPECTRA

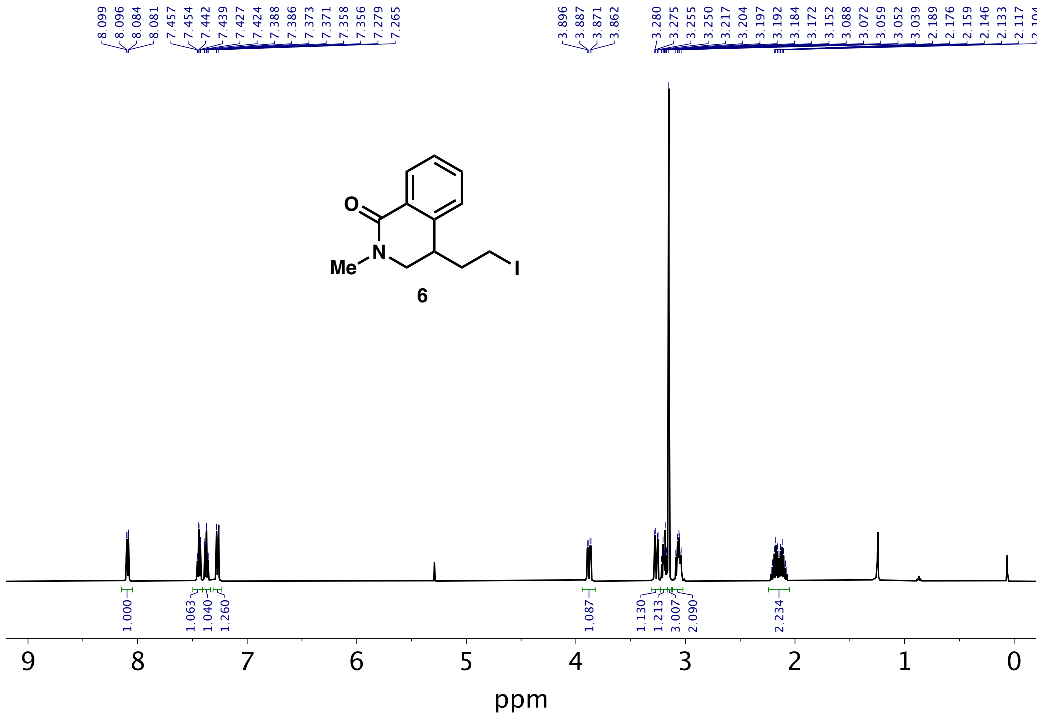

Figure S6. ^{1}H NMR (500 MHz, $CDCl_3$, 298 K) of **6**.

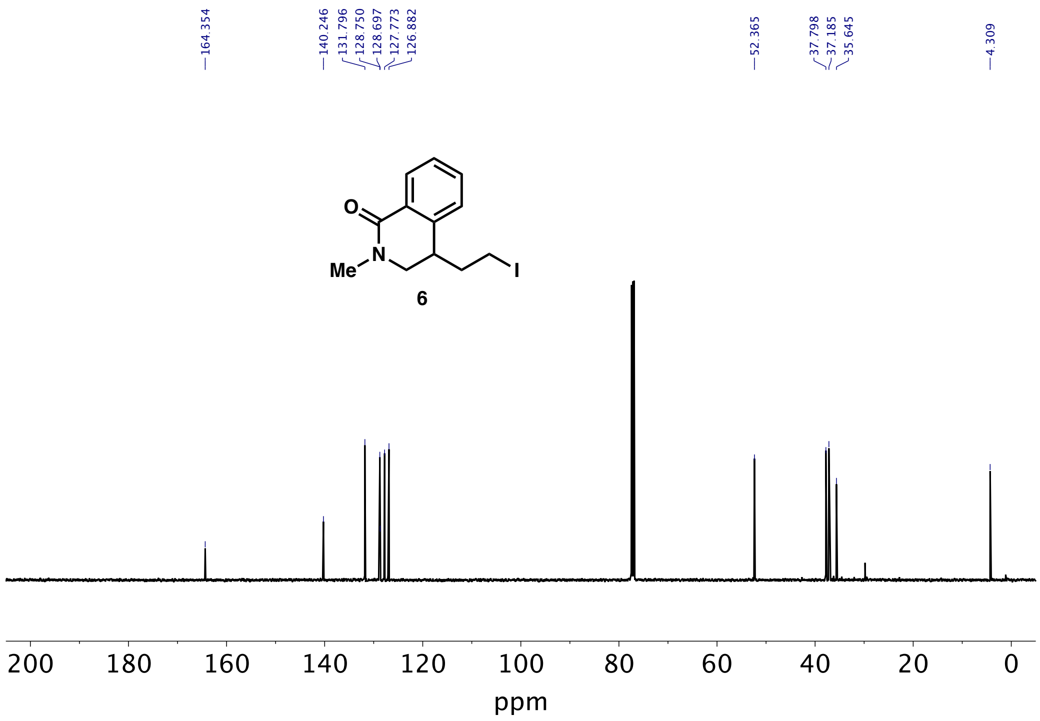

Figure S7. $^{13}C\{^{1}H\}$ NMR (126 MHz, $CDCl_3$, 298 K) of **6**.

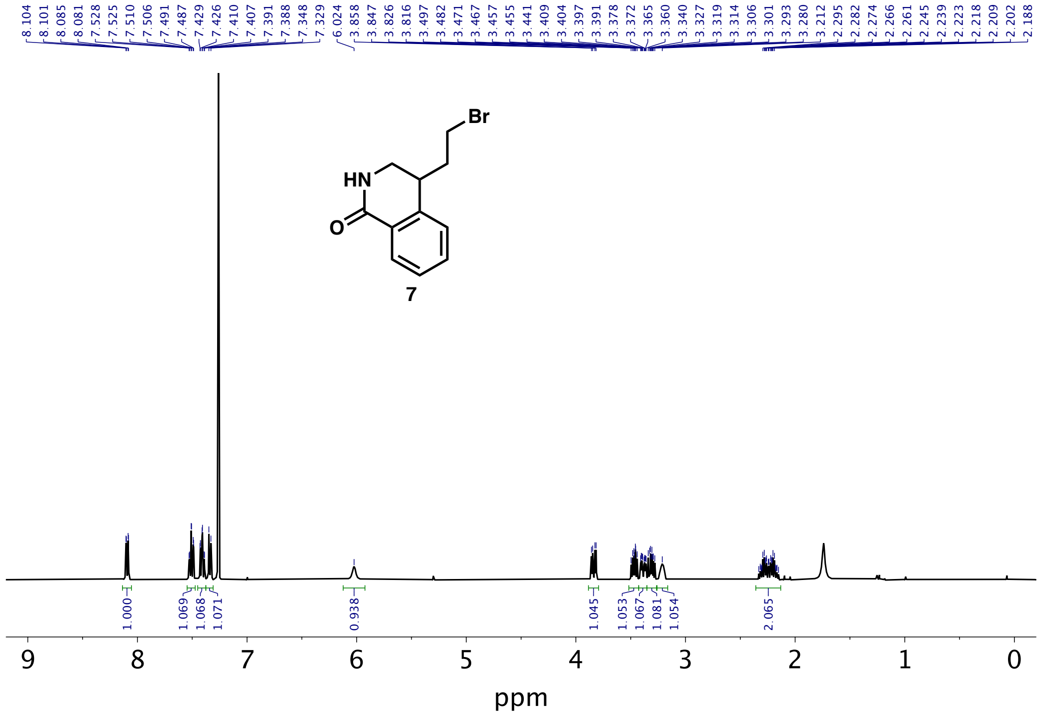

Figure S8. ^{1}H NMR (400 MHz, $CDCl_3$, 298 K) of **7**.

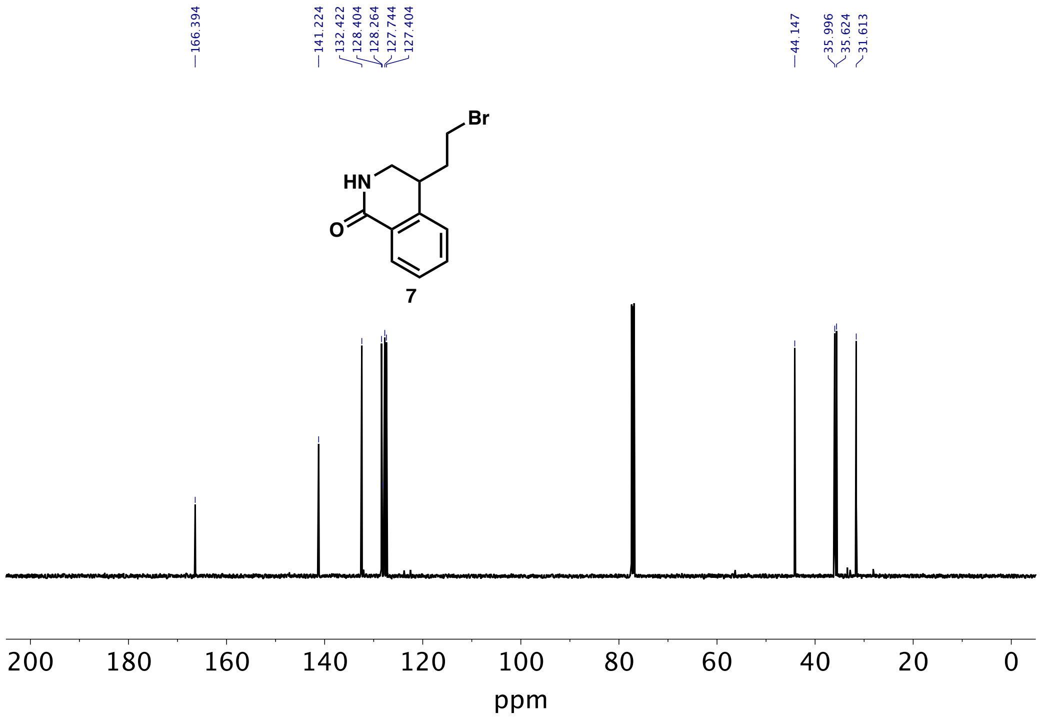

Figure S9. ^{13}C{^{1}H} NMR (126 MHz, $CDCl_3$, 298 K) of **7**.

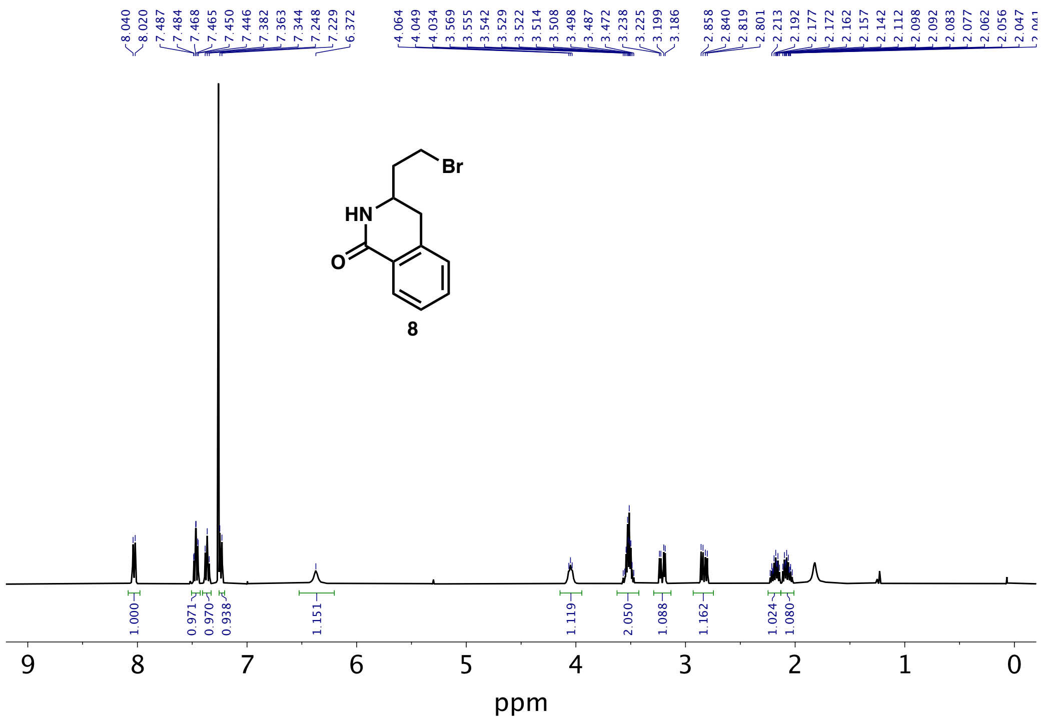

Figure S10. ^{1}H NMR (400 MHz, $CDCl_3$, 298 K) of **8**.

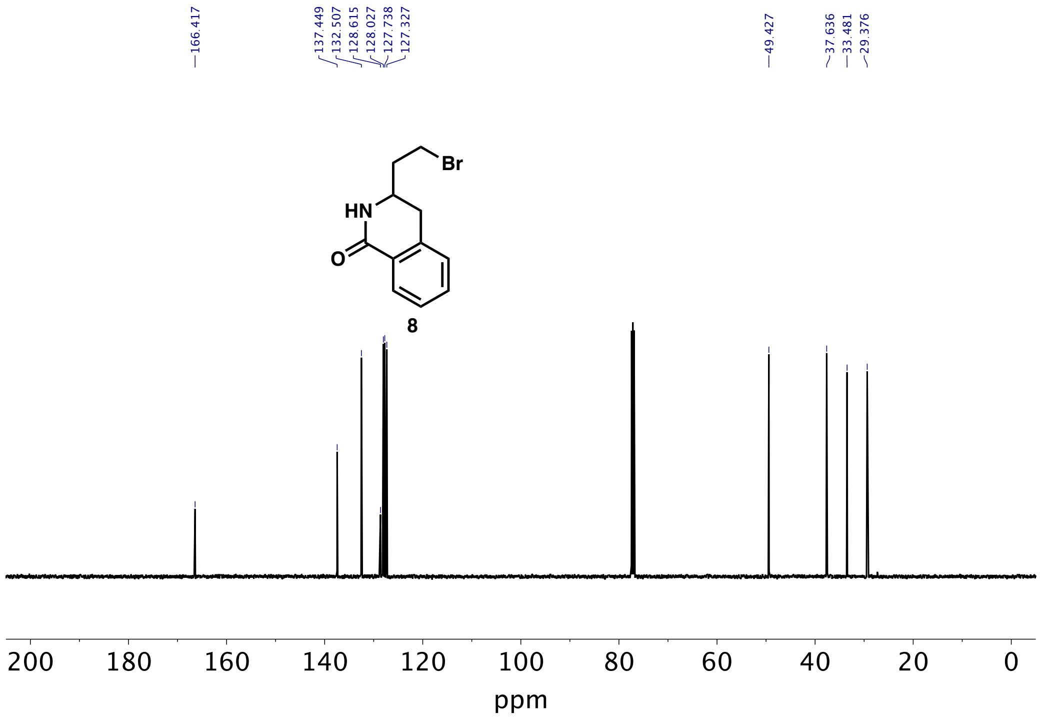

Figure S11. $^{13}C\{^1H\}$ NMR (126 MHz, $CDCl_3$, 298 K) of **8**.

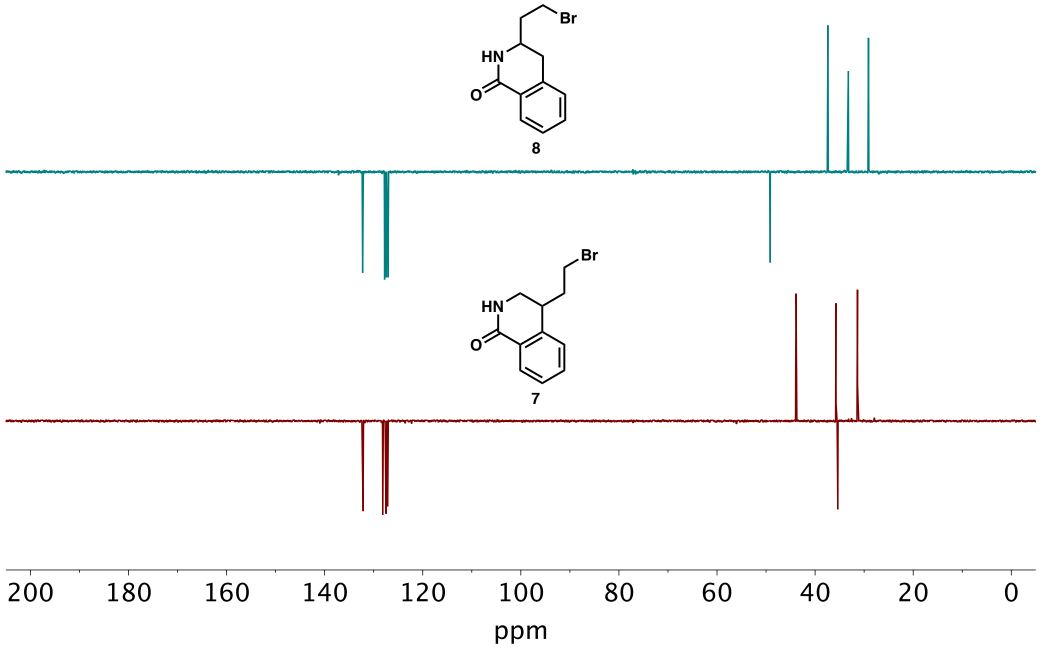

Figure S12. Stacked DEPT-135 spectra (126 MHz, $CDCl_3$, 298 K) of **7** and **8**.

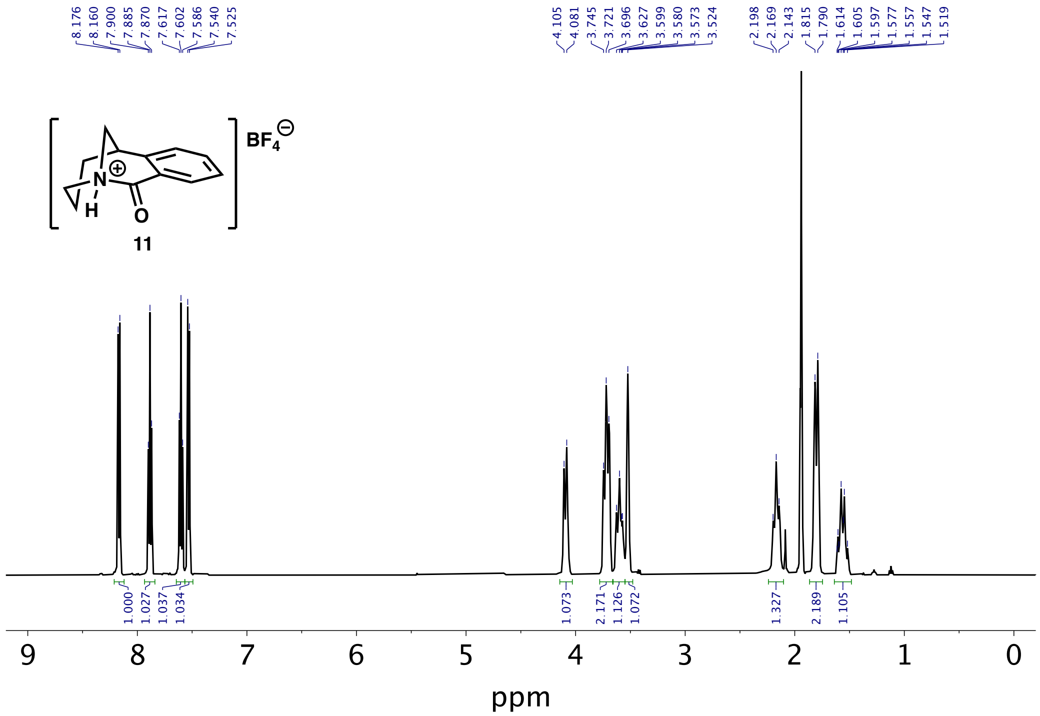

Figure S13. ^{1}H NMR (500 MHz, CD_3CN, 298 K) of **11**.

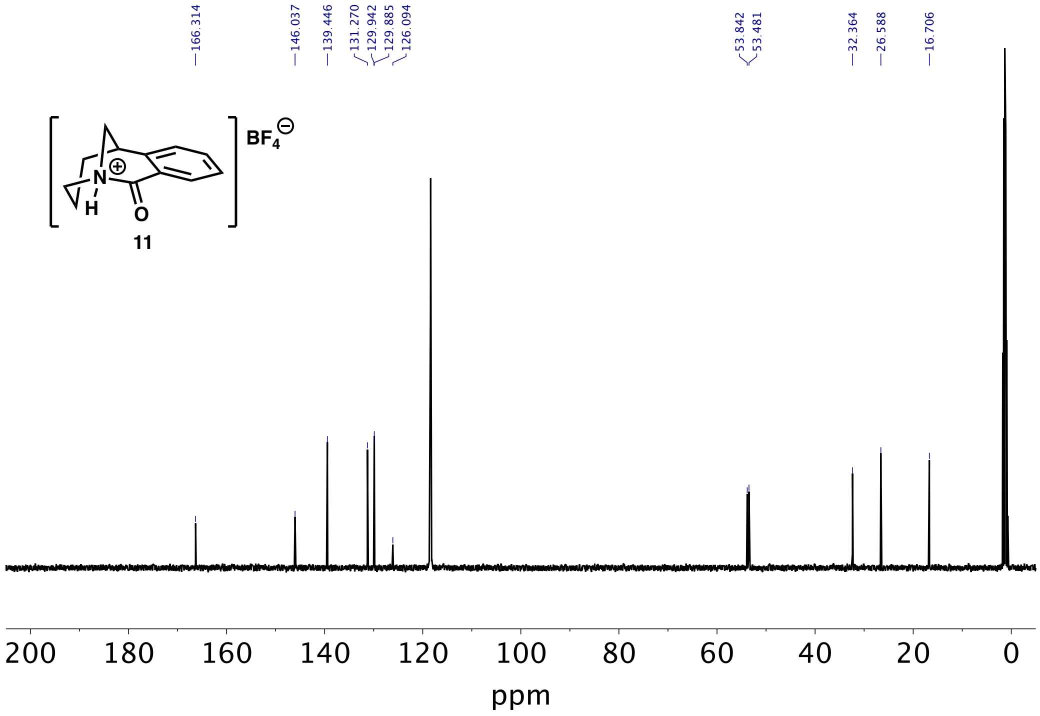

Figure S14. $^{13}C\{^{1}H\}$ NMR (101 MHz, CD_3CN, 298 K) of **11**.

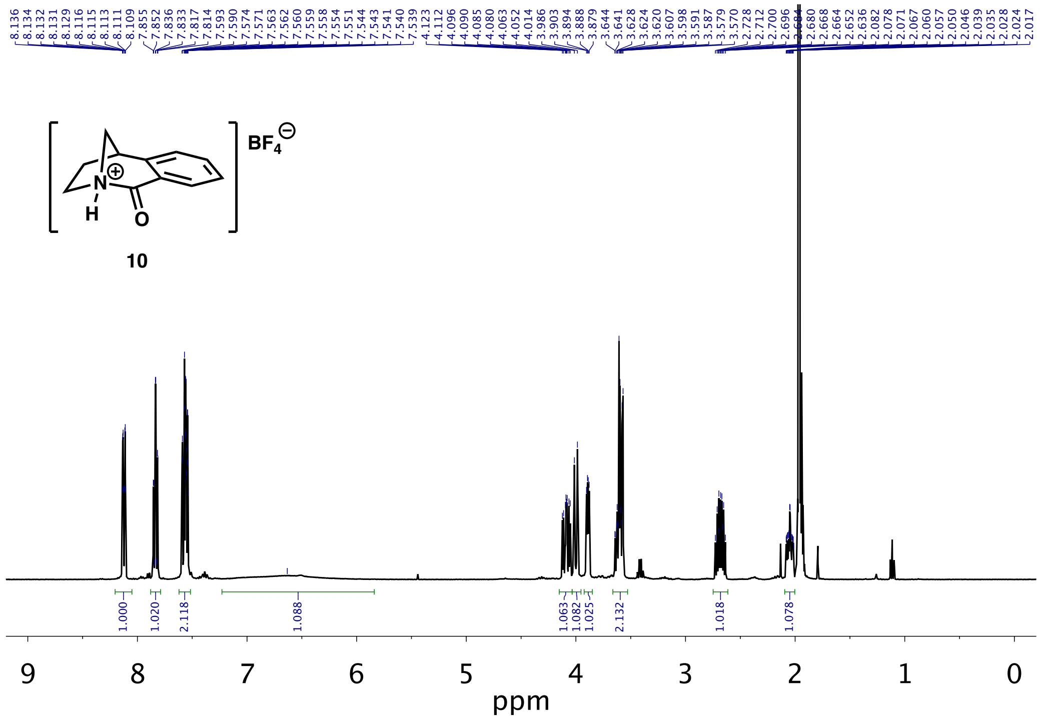

Figure S15. ^{1}H NMR (400 MHz, CD_3CN, 298 K) of **10**.

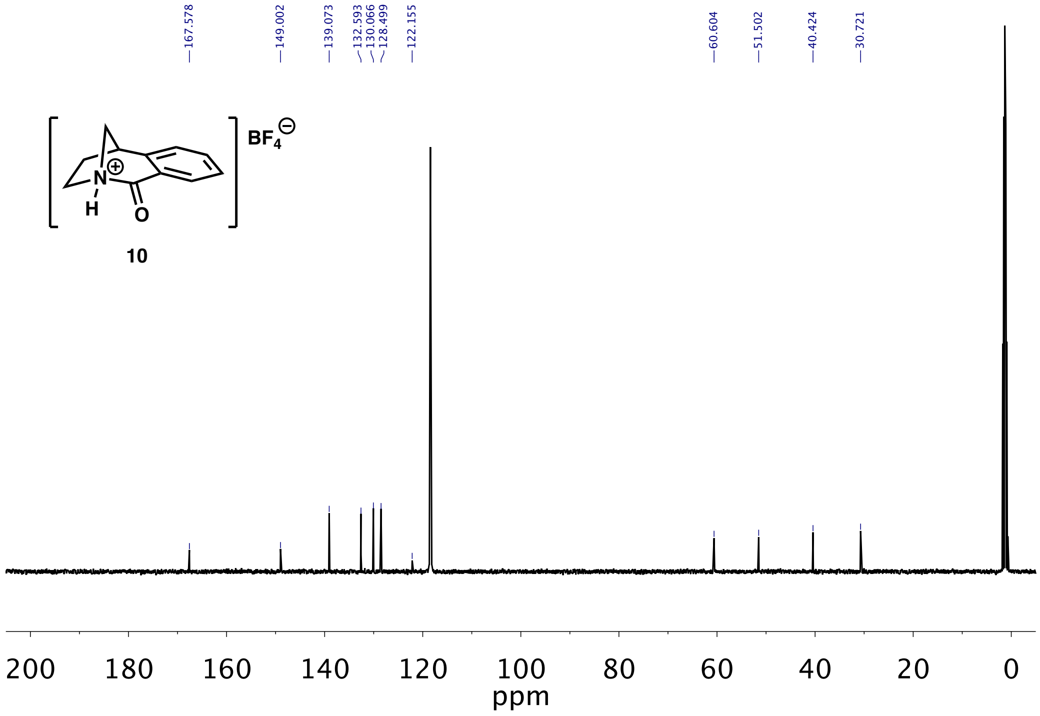

Figure S16. $^{13}C\{^{1}H\}$ NMR (101 MHz, CD_3CN, 298 K) of **10**.

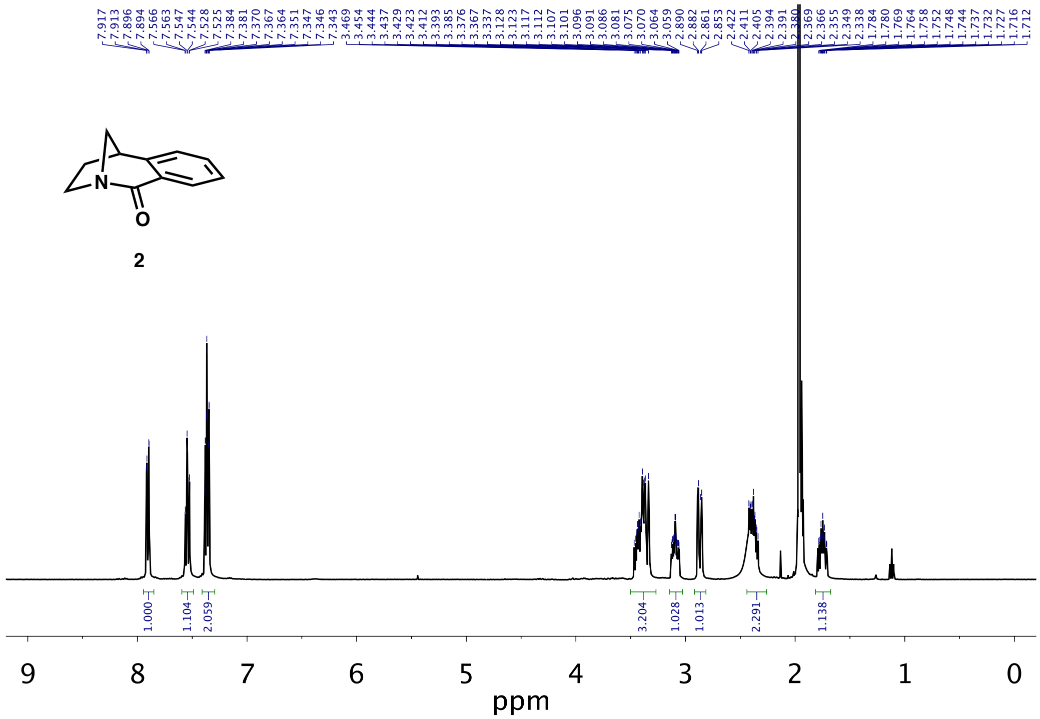

Figure S17. ^{1}H NMR (400 MHz, CD_3CN, 298 K) of **2** (from *in situ* neutralization of **10** using Cs_2CO_3).

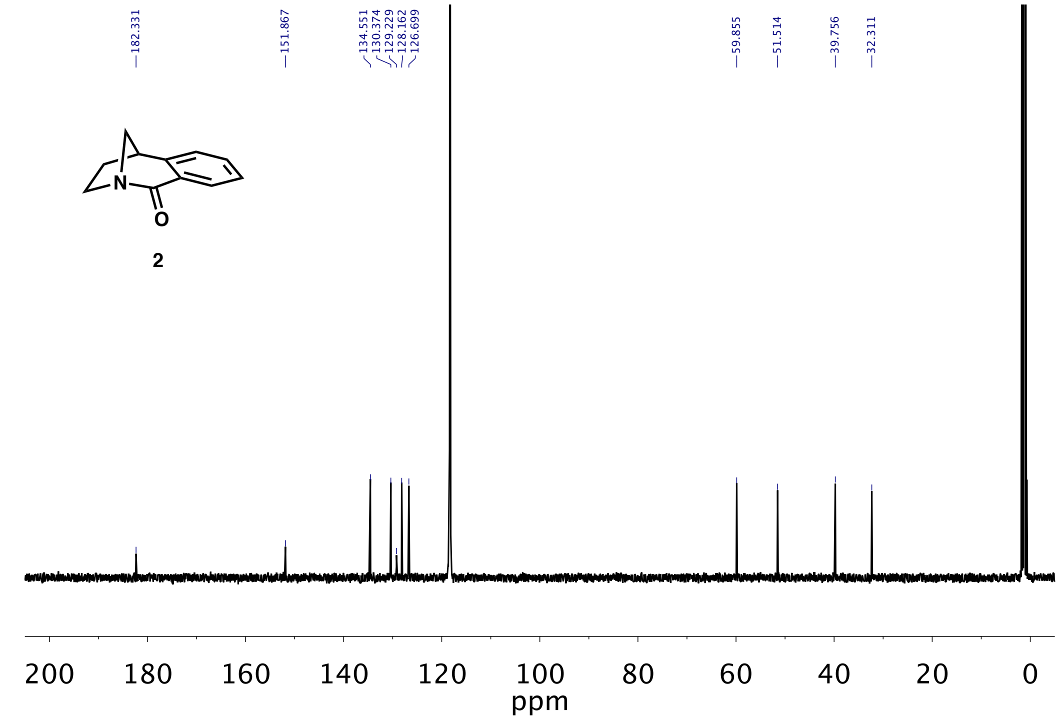

Figure S18. $^{13}C\{^1H\}$ NMR (101 MHz, CD_3CN, 298 K) of **2** (from *in situ* neutralization of **10** using Cs_2CO_3).

REFERENCE

(1) Hu Feng; Lalancette Roger; Szostak Michal. Structural Characterization of N-Alkylated Twisted Amides: Consequences for Amide Bond Resonance and N−C Cleavage. *Angew. Chem. Int. Ed.* **2016**, *55* (16), 5062–5066.

(2) Fu, L.; Xu, M.; Yu, J.; Gutekunst, W. R. Halide-Rebound Polymerization of Twisted Amides. *J. Am. Chem. Soc.* **2019**, *141* (7), 2906–2910.

(3) Parrish, R. M.; Burns, L. A.; Smith, D. G. A.; Simmonett, A. C.; DePrince, A. E. I.; Hohenstein, E. G.; Bozkaya, U.; Sokolov, A. Yu.; Di Remigio, R.; Richard, R. M.; Gonthier, J. F.; James, A. M.; McAlexander, H. R.; Kumar, A.; Saitow, M.; Wang, X.; Pritchard, B. P.; Verma, P.; Schaefer, H. F. I.; Patkowski, K.; King, R. A.; Valeev, E. F.; Evangelista, F. A.; Turney, J. M.; Crawford, T. D.; Sherrill, C. D. Psi4 1.1: An Open-Source Electronic Structure Program Emphasizing Automation, Advanced Libraries, and Interoperability. *J. Chem. Theory Comput.* **2017**, *13* (7), 3185–3197.

(4) Szostak, R.; Szostak, M. Tröger's Base Twisted Amides: High Amide Bond Twist and N-/O-Protonation Aptitude. *J. Org. Chem.* **2019**, *84* (3), 1510–1516.

(5) Szostak, R.; Aubé, J.; Szostak, M. Determination of Structures and Energetics of Small- and Medium-Sized One-Carbon-Bridged Twisted Amides Using Ab Initio Molecular Orbital Methods: Implications for Amidic Resonance along the C–N Rotational Pathway. *J. Org. Chem.* **2015**, *80* (16), 7905–7927.

(6) Greenberg, A.; Moore, D. T.; DuBois, T. D. Small and Medium-Sized Bridgehead Bicyclic Lactams: A Systematic Ab Initio Molecular Orbital Study. *J. Am. Chem. Soc.* **1996**, *118* (36), 8658–8668.

(7) Greenberg, A.; Venanzi, C. A. Structures and Energetics of Two Bridgehead Lactams and Their N- and O-Protonated Forms: An Ab Initio Molecular Orbital Study. *J. Am. Chem. Soc.* **1993**, *115* (15), 6951–6957.

(8) Xu, M.; Paul, M. K.; Bullard, K. K.; DuPre, C.; Gutekunst, W. R. Modulating Twisted Amide Geometry and Reactivity Through Remote Substituent Effects. *J. Am. Chem. Soc.* **2021**, *143* (36), 14657–14666.
